# Autonomous thermodynamically informed database generation for machine-learned interatomic potentials and application to magnesium

**DOI:** 10.1038/s41524-025-01903-z

**Published:** 2025-12-17

**Authors:** Vincent G. Fletcher, Albert P. Bartók, Livia B. Pártay

**Affiliations:** 1 Department of Physics, University of Warwick, Coventry, UK; 2 Warwick Centre for Predictive Modelling, School of Engineering, University of Warwick, Coventry, UK; 3 Department of Chemistry, University of Warwick, Coventry, UK

**Keywords:** Chemistry, Materials science, Mathematics and computing, Physics

## Abstract

We propose a novel approach for constructing training databases for Machine-Learned Interatomic Potential (MLIP) models, specifically designed to capture phase properties across a wide range of conditions. The framework is uniquely appealing due to its ease of automation, its suitability for iterative learning, and its independence from prior knowledge of stable phases, avoiding bias towards pre-existing structural data. The approach uses Nested Sampling (NS) to explore the configuration space and generate thermodynamically relevant configurations, forming the database which undergoes ab initio Density Functional Theory (DFT) evaluation. We use the Atomic Cluster Expansion (ACE) architecture to fit a model on the resulting database. To demonstrate the efficiency of the framework, we apply it to magnesium, developing a model capable of accurately describing behaviour across pressure and temperature ranges of 0–600 GPa and 0–8000 K, respectively. We benchmark the model’s performance by calculating phonon spectra and elastic constants, as well as the pressure-temperature phase diagram within this region. The results showcase the power of the framework to produce robust MLIPs while maintaining transferability and generality, for reduced computational cost. UK Ministry of Defence ©Crown Owned Copyright 2025/AWE

## Introduction

The development and application of machine learning-based interatomic potentials has become widespread in atomistic simulations, offering near ab initio accuracy at a fraction of the computational cost. The past decade has seen rapid growth in the development of MLIPs, with the creation of different descriptors^1–15^, the use of various architectures^6,16–19^, and the proposal of diverse workflows^20–22^, machine learning based models have been tailored for a wide range of materials.

While the underlying architectures of MLIPs can differ significantly, they all rely on the quality and representativeness of the training dataset. Regardless of the specific framework, the accuracy and transferability of these models are fundamentally tied to the database used for their development. This highlights a key avenue for advancing MLIPs: refining the construction of training datasets. In this study, we tackle the challenge by creating a procedure for constructing robust and transferable databases that capture thermodynamically relevant behaviour under a wide range of conditions, applicable to any machine learning frameworks.

Functionally, MLIPs replace computationally expensive ab initio calculations with an approximate solution. Balancing model complexity and accuracy with computational expense, MLIPs are typically created to operate in narrow regions of phase space and are designed for each study by training on samples of the ab initio Potential Energy Surface (PES). However, due to the high dimensionality of the PES, and the high cost of ab initio calculations, it is expensive to sample and unclear how to do so efficiently. With access to vast databases and resources from years of computational studies, recent developments are pushing these frameworks to the limits by creating so-called foundation models, with a focus on sensible predictions across extensive phase and chemical space, but with reduced accuracy compared to purpose-built potentials^23–33^. These models can then be used as a foundation for fine-tuning by either: creating databases for a more specific application;^34–36^ or by refitting part of the model, for greater accuracy in a specific region of phase space^37–39^. Creating databases suitable to represent a high diversity of conditions has its particular challenges. Large databases come with more ab initio evaluations, and more data means models become more expensive to fit. Additionally, large models are required to accurately reproduce a high diversity of properties which increases the cost of model evaluations and further increases the cost of fitting. Another point of consideration is the importance assigned to individual sample points during the fitting procedure, to avoid artificially prioritising the accuracy of a specific phase or property, the weight associated with types of samples in the database must be taken into account during fitting. These points highlight the importance of the density of samples within the database and, by extension, the method by which these samples are collected.

MLIP database construction is not yet standardised, and the process of constructing a database depends both on the desired application and the available information. If phases of the material are known, initial databases are usually constructed algorithmically, based on heuristics, such as: ground state structures, and their strained versions; surface slabs and defect configurations; and finite temperature ab initio Molecular Dynamics (AIMD)^40^ snapshots^41–45^. This strategy works suitably well in most cases, but it is computationally demanding, requires some prior knowledge of the material’s properties, and has an inherent bias towards known and expected structures - meaning that important configurations and phases can be missed^46^. Multiple procedures and improvements have been proposed to deal with different aspects of this problem. For workflows that rely on AIMD to generate databases, a hybrid approach can be used, whereby energies and forces are predicted using a MLIP until some threshold of prediction uncertainty is crossed, at which point ab initio evaluations are performed, the model retrained, and the propagation continued^47^. This type of hybrid AIMD approach, with the goal of accelerating the process of obtaining Molecular Dynamics (MD) trajectories, can provide a 20–500 times speed-up depending on the model, phase, and material complexity. While MD simulations are a powerful tool for suggesting thermodynamically relevant samples from within a bound region of the PES, they are inefficient at locating a broad range of phases and rely entirely on the chosen thermodynamic conditions. Additionally, samples are notoriously hard to decorrelate and with higher energy simulations they become more difficult to stabilise, requiring smaller time-steps to conserve energy. To help alleviate a few of these drawbacks, some approaches run MD simulations with a MLIP but with an additional term added to the predicted energy to bias dynamics towards unseen configurations. In doing so, MD trajectories frequently sample unseen regions of the PES, meaning ab initio evaluations can be limited to configurations that are not already represented in the training data^48,49^. This targetted approach to sampling attempts to increase sample diversity and reduce redundancy in the training database and these principles have also been applied without using MD to drive the sampling. By leveraging local atomic environment descriptors, a measure of similarity between environments can be calculated, and sampling can then be directed to maximise descriptor diversity thereby reducing sample redundancy and promoting diversity^50,51^. While new methods that target descriptor diversity are likely to produce representative databases, the goal of structure searching algorithms, developed for over 20 years now, has been to sample the entire structure space for stable structures, outputting a highly diverse set of relevant structures.

This need for diversity in the training data has motivated the development of methods which use structure generation algorithms to produce relevant configurations, such as Universal Structure Predictor: Evolutionary Xtallography (USPEX) or ab initio random structure search (AIRSS)^52,53^. Such methods generate structures of a given number of atoms randomly under very general constraints – such as a maximum volume or minimum interatomic distance or within a certain space group – and a gradient based structure relaxation is used to find local minima of the ab initio or MLIP PES. The resulting minima can then be perturbed to provide gradient information, and a MLIP trained on the resulting set of configurations^22,44,54,55^. Another MD-independent method includes targetted sampling along high symmetry phonon modes, which has been applied to titanium and titanium alloys^21,56^. These methods are highly efficient at generating large datasets with less oversight, relative to MD based procedures, but the advantage of MD simulations is that samples are drawn as a function of the Boltzmann distribution, giving a higher weight to configurations – through increased sample density – with the highest free energy or thermodynamic relevance. Any sampling technique that is not based on equilibrium thermodynamics has to guide sample density to ensure all relevant phases are properly represented in the database.

In our current work, we propose the use of the NS algorithm to combine the advantages of Random Structure Search (RSS) and MD based approaches, allowing the creation of a procedure that is easy to automate, generates atomic configurations across all relevant phases, free from preconceived ideas about the materials’ properties, while taking into account the thermodynamic behaviour of the material.

The NS algorithm generates configurations as a function of their thermodynamic relevance from the ideal gas through to the ground state structure^57–61^. Each phase within the database is – by nature of the sampling – inherently weighted as a function of the phase-space volume it occupies. Hence, the created dataset contains structural and thermodynamic information under all thermodynamically relevant conditions representative of the entire configuration space of the material. Since only the most thermodynamically relevant configurations undergo high-cost ab initio evaluation, our procedure decreases the cost of evaluating a database. Additionally, since the number of samples representing a basin is based on the associated phase space volume, multiple databases can be trivially combined together. This allows simple extension of the training database without the need to discard existing data or change the inherent weighting associated with specific phases or energies.

As a test system for our procedure, we chose elemental magnesium. Magnesium has been studied extensively across a wide pressure range (0−100 GPa) both experimentally^62–66^, and by ab initio calculations^67–71^, providing us with substantial benchmark data. Furthermore, extreme pressure phases have also been predicted by simulations, up to 1.6 TPa, with some recent experimental results as confirmation^72–74^. At 0 K, the hexagonal close-packed (HCP) structure is the ground state crystalline phase up to ~53 GPa, at which point a transition to the body-centred cubic (BCC) phase is observed. BCC remains the stable solid phase up to around 456 GPa, when a transition to the face-centred cubic (FCC) phase is predicted. This relatively straightforward phase behaviour provides a typical scenario an interatomic potential model should be able to capture, while the vast pressure range represents a challenge for efficient database building. The MLIP for magnesium fitted by Ibrahim et al.^75^ used the database generation method based on structural templates, resulting in a high quality model reliable up to 70 GPa and 4000 K trained on 40,000 individual DFT reference calculations resulting in 738,705 atomic environments.

Although the phase diagram of magnesium has been extensively investigated, some phase transitions and regions with interesting properties remain debated and this is where a high-accuracy MLIP can be critical to enhance the extent of current sampling capabilities and further our understanding of the atomic level properties of magnesium. Between 5 and 20 GPa in the high temperature solid region immediately below the melting line, the thermodynamically stable phase is debated^66^. Experimental measurements suggest that an additional crystalline structure, with characteristics similar to that of the double-hexagonal close-packed (dHCP) structure, emerges but to our knowledge its precise structure has not yet been identified^66^.

## Results and discussion

### Initiating and expanding a database

**Fig. 1:**
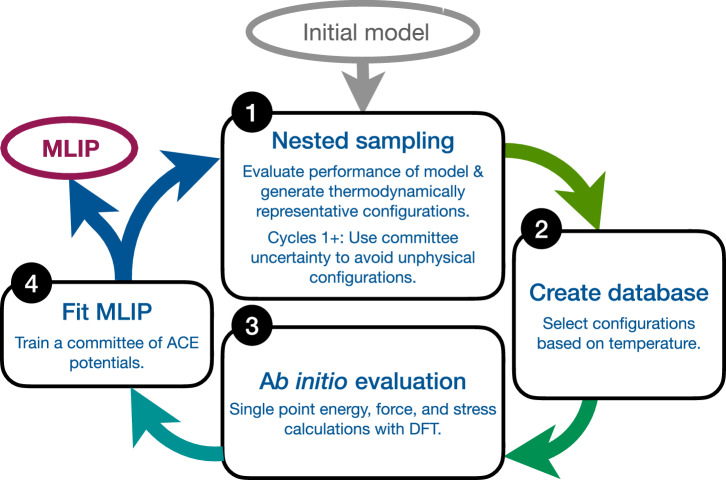
Schematic workflow of the iterative potential fitting process. The cycle starts with generating configurations from NS. In the zeroth cycle this is done using an arbitrary initial potential, an EAM in the current work. A database is autonomously constructed, guided by the thermodynamic information and samples generated by NS. The database undergoes DFT evaluation and an MLIP is (re)fitted, acting as a new input to the next cycle for further refinement if needed. We repeated this cycle 5 times.

Our method of creating the database and fitting the MLIP consists of four key stages, summarised schematically in Fig. 1, with the key parameters for each stage given in Table 1. We refer to the produced ACE models through notation reflecting the cycle it was used in and the order and degree of the ACE model. For example, the order 4, degree 14 ACE model used in cycle 2, will be referred to as C2 O4 D14. First, a NS calculation is performed using a classical interatomic potential, producing samples across all phases of the material; we then use the thermodynamic properties calculated from NS to produce a more selective database; this refined database then undergoes ab initio DFT evaluation; finally an ACE MLIP is fitted to the evaluated database. We define two independent procedures for initiating or expanding a database, and since the initial cycle is performed independent of an MLIP, we label this cycle the zeroth cycle. The specific details of these stages are discussed in the following.

**Table 1:** Key parameters used across the active learning cycles

	Nested sampling				Database Building		Fitting	
Cycle	Model used	Atoms	Pressure range	Max STD	Temp. range	Samples added	Sample weight	Model produced
			[GPa]	[meV/at]	[K]			
0	EAM	16	0, 1, [5–45,5]	N/A	200–3000	1100	Equal	C1 O4 D14
1	ACE: C1 O4 D14	16	0, 1, [5–45,5], [60–600,20]	62.5	Lowest	39	Equal	C2 O4 D14
2	ACE: C2 O4 D14	16	0, 1, [5–45,5], [60–600,20]	62.5	200–8000	2801	Equal	C3 O4 D14
3	ACE: C3 O4 D14	16	0, 1, [5–45,5], [60–600,20]	–	0–1000	390	*α* = 0.1	C4 O4 D14
4	ACE: C4 O4 D14	8	0, 1, [5–45,5], [60–600,20]	–	Lowest	3900	*α* = 0.1	C5 O4 D18

We define the critical parameters used during: the NS exploration, the selection of configurations to be added to the training database, and the subsequent MLIP fitting. The label for the models produced refer to the cycle the potential will be used in, and the order and degree of the ACE model.

### Nested sampling

The NS method can efficiently sample high dimensional spaces and evaluate integrals of functions defined in such spaces^57,58,61^. NS has been used in a materials context to explicitly evaluate the partition functions of atomistic systems at arbitrary conditions^60^. With the full partition function, one has access to thermodynamic response functions and hence is able to determine the location of phase transitions and characterise properties of the material as a function of temperature during a post-processing step.

In general, the algorithm works by sampling the entire phase space of the material iteratively, starting from the gas phase towards the solid phase, generating configurations proportional to the phase-space that they occupy, without any prior knowledge of specific phases or structures. The power of NS has been demonstrated with numerous materials, from atomic clusters^60,76,77^, to soft-matter potentials^78,79^, and metallic systems^80,81^.

Here we briefly describe the NS technique, as employed in the current work, sampling bulk phase configurations at constant pressure, and using total-enthalpy Hamiltonian Monte Carlo to modify configurations^82^. The algorithm can be described by the following six steps:

1. Generate *K* random configurations of *N* atoms in a cell, with a maximum volume defined by randomly generated cell vectors. These configurations are referred to as walkers.
2. Calculate the enthalpy, *H*, of each walker and eliminate the one with the highest enthalpy.
3. From the pool of remaining walkers, randomly select one and clone it.
4. Perform a series of random moves on the cloned walker: cell distortions and short *NVE* MD simulations. This process is referred to as ‘walking’, and the number of moves is referred to as the ‘walk length’.
5. Calculate the enthalpy of the walked clone. If it is lower in enthalpy than its parent, it is accepted as a new walker, if not it is rejected and step 3 onwards is repeated.
6. Once a new walker has been accepted, the procedure from step 2 is repeated.

The key result is that after step 2 of iteration *i*, the initial phase space volume, Γ_0_, has been reduced by a known factor to Γ_*i*_, provided the sampling is uniformly random.

1 $${\Gamma }_{i}={\Gamma }_{0}\exp \left(-\frac{i}{K}\right)$$

With the change in phase-space volume at each iteration known, one can exactly compute the partition function, *Z*.

2 $$Z(\beta )\approx \sum _{i}\left({\Gamma }_{i}-{\Gamma }_{i+1}\right){e}^{-\beta {H}_{i}}$$

Therefore one can compute any equilibrium property of interest, *O*, as a function of the thermodynamic *β*, after only one sampling procedure.

3 $$O(\beta )\approx \frac{{\sum }_{i}{O}_{i}\left({\Gamma }_{i}-{\Gamma }_{i+1}\right){e}^{-\beta {H}_{i}}}{Z(\beta )}$$

The challenge of NS lies in producing random samples uniformly from a constantly shrinking sample space. In practice, steps 3–5 enable this by generating a new sample configuration via a random walk - which decorrelates the clone of a randomly selected existing configuration. These steps account for the majority of the computational cost of the algorithm; thus, in total, NS requires on the order of 10^8^ energy evaluations for a typical system described in the current work, most of which are spent on the cloning and walking procedure. When using NS to calculate the pressure-temperature phase diagram of the final ACE potential, we took advantage of the recently proposed extension to the sampling, replica-exchange-NS^83^, to allow better resolution of low temperature solid-solid phase transitions.

### Initiating the training database

To generate the initial magnesium database in cycle 0, a series of NS calculations were carried out using 16-atom cells, with the interaction modelled by the Embedded Atom Method (EAM) potential developed for magnesium by Wilson et al.^84^ at eleven different pressure values: 0 GPa, 1 GPa, and every 5 GPa between 5 and 45 GPa inclusive. This model underestimates the BCC melting temperature considerably, and it also incorrectly predicts a HCP to FCC solid-solid transition, as shown in Supplementary Fig. 1. These shortcomings provide an ideal scenario for evaluating the ability of our training procedure to correct or expand an existing model. While we have chosen this particular EAM model to generate initial configurations, a more approximate (e.g. Lennard-Jones) or a more advanced model (e.g. foundation MLIP) could have been selected as well. After each NS run, the temperature-dependent enthalpy curve was calculated using Eq. (3), providing the temperature at which each sampled configuration has the highest probability to occur.

**Fig. 2:**
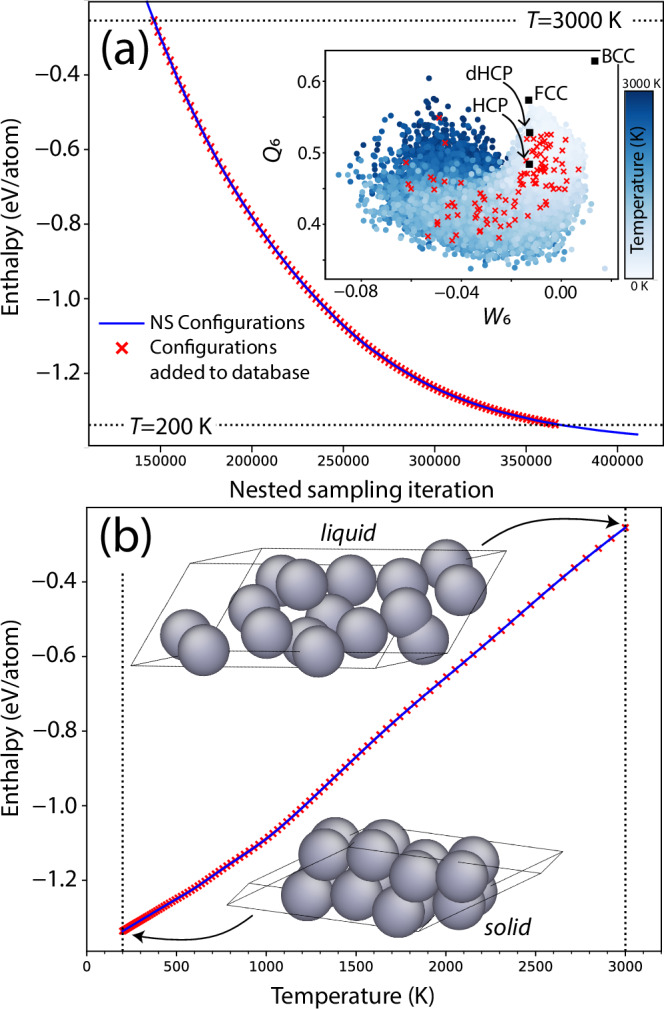
The enthalpy of configurations generated during NS in cycle 0 at 1 GPa. **a** Shows the enthalpy of the samples plotted as a function of NS iterations, with the inset showing the average *Q*_6_ and *W*_6_ Steinhardt bond order parameters of the configurations, coloured by their associated temperature. **b** Shows the enthalpy of samples plotted as a function of temperature, with snapshots of the highest and lowest enthalpy configurations selected for training shown. The 100 configurations, equally spaced in iteration number, that were added to the database are marked by red crosses. In cycle 0 all samples were generated using the EAM model.

In order to automatically exclude the least relevant gas phase configurations and select a diverse range of samples from the high-temperature liquid phase to low-temperature crystalline phases, we defined a temperature range of 200–3000 K to select configurations from. This range generously encompasses the melting line across the entire sampled pressure range (the melting temperature of the EAM model is 1051 K and 1623 K at 1 GPa and 45 GPa, respectively). From this range, 100 configurations were selected, equally spaced in iteration number, as shown in Fig. 2 for the 1 GPa sampling. This provided a wide distribution of samples of and around the relevant potential energy basins as shown in Fig. 2 through the distribution of samples across the *W*_6_ and *Q*_6_ Steinhardt bond order parameters^85^. This automatic selection was repeated at each pressure, resulting in a total number of 1100 16-atom configurations (17,600 atomic environments) collected to construct the initial database. These configurations underwent DFT evaluation and were then used to train our first ACE potential. This model (C1 O4 D14) was then used in the consecutive training cycle: cycle 1.

### Expanding the range

It is trivial to repeat the previously described procedure, performing NS calculations using the ACE model to gather more samples and expand the training set as necessary, a typical example of active learning. However, in the early stages of this process, or when the local atomic environments deviate significantly from those represented in the training data, MLIPs can behave unpredictably. This often manifests as so-called *holes* in the PES where the model assigns unfeasibly low energies to certain structures, typically those with unphysically short interatomic distances. Holes are usually associated with sudden and drastic changes in the energies and forces, leading to serious issues during geometry optimisation or MD simulations. Such behaviour is a common challenge in MLIPs, although they can remain undetected by sampling techniques which typically explore the phase space in near-equilibrium conditions.

In contrast, due to its exhaustive sampling strategy, NS is highly effective in uncovering these problematic regions (with increasing the number of walkers, and thus the resolution of the sampling, holes with smaller phase space volume become possible to identify). While this capability is desirable for identifying flaws in the PES and improving the training dataset in a targeted way, these configurations can interrupt the NS algorithm as, once found, they dominate the rest of the sampling iterations due to their low energy. Simply avoiding these configurations by applying a minimum distance cutoff or similar heuristics is not straightforwardly generalisable. For example, when describing high pressure or temperature behaviour, *physical* short interatomic distances occur, which should not be removed. Adding such configurations to the training data is unworkable due to the unfeasibility of ab initio calculations of such configurations, which typically fail numerically due to core overlaps of the pseudopotential.

**Fig. 3:**
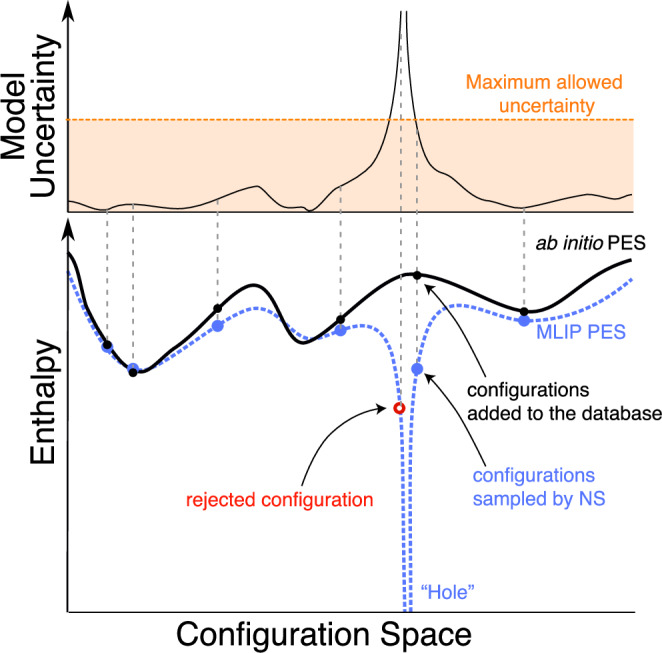
Schematic representation of sampling with a model committee STD restriction. By sampling with the model committee STD restriction, the walkers avoid becoming trapped in holes of the PES during NS. Solid black and dashed blue lines represent the target ab initio PES and the MLIP PES respectively. Blue circles indicate configurations generated during the sampling, with corresponding black circles showing the same configurations after evaluation by DFT which are thereafter added to the database for MLIP training. Upper panel demonstrates the corresponding uncertainty of the model, with the orange dashed line indicating the limit, above which samples are rejected, shown by the red circle on the PES.

In order to employ NS in the presence of the holes, we utilise the uncertainty quantification measure provided by the ACE committee framework, demonstrated schematically in Fig. 3. Configurations corresponding to PES holes contain atomic environments unseen during the fitting procedure, hence, the energy estimate of such environments have a high Standard Deviation (STD). We found this metric to be significantly higher than for any other configuration in any other phase across the entire pressure range, and thus it is suitable to identify the PES holes, independent of pressure and temperature. If the STD of the committee is incorporated into the sampling as an acceptance criteria, the exploration of unfamiliar basins can be tuned to stop before the samples become unlike anything physical seen in the database which would cause the subsequent ab initio calculations to fail.

In cycle 1, the first step in expanding the sampled pressure range is to run NS across the entire pressure range of interest – 0–600 GPa – with an additional acceptance criteria introduced during the random walk to generate new samples (step 4 of the algorithm described in the Nested Sampling Section): after each proposed move we evaluate the committee STD in the prediction of the total energy, and if this value is above 62.5 meV/atom, the move is rejected. Additionally, since the presence of PES holes makes the prediction of thermodynamic properties and temperature unreliable, which is the basis of the stopping criteria, a NS calculation is also terminated if 90% of the walkers have a total energy committee STD value above 60 meV/atom. We found this criteria is only met when, during the sampling, all the walkers approach unphysically low energy regions and become immobilised. Only the final configuration from these simulations were subject to DFT evaluation and were subsequently added to the database. Thus, only 39 16-atom configurations were added during this cycle, resulting in a total of 1139 16-atom configurations in the training database at this stage.

**Fig. 4:**
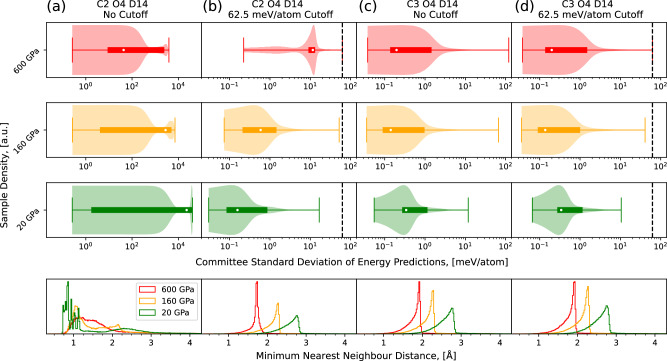
Distribution of committee STD of samples and minimum interatomic distance within those samples, across cycles, across pressure, with and without the maximum STD restriction enabled. We show how the committee STD (top three rows) and minimum bond length (bottom row) of samples changes across pressures (red: 600 GPa, orange: 160 GPa, green: 20 GPa), across cycles (cycle 2: column (**a**) and (**b**), cycle 3: column (**c**) and (**d**)) both with (column (**b**) and (**d**)) and without (column (**a**) and (**c**)) the committee STD restriction, shown by the black dashed line. We highlight the unphysically short bond lengths and substantial STD values in column (**a**) and show how the STD restriction, applied in column (**b**), corrects this behaviour. We also highlight the minimal effect of the STD restriction when physical samples are produced by comparing columns (**c**) and (**d**).

In the cycle 2, we repeated the procedure across the entire pressure range again. In case of the 1 GPa sampling, the run stopped due to high committee STD, and only the final configuration was evaluated with DFT and added to the database. All the other NS runs terminated when the estimated temperature reached 200 K and from the runs in the extended pressure range (60+GPa), 100 configurations were chosen that were equally spaced in iteration number, within an extended temperature range of 200–8000 K, compared to cycle 0. After this cycle, a total of 2801 16-atom configurations were added to the database. To illustrate the use and necessity of the model uncertainty criteria, in Fig. 4 we show the committee STD across NS runs at 20, 160, and 600 GPa, both with and without the STD cutoff. When attempting to perform NS in cycle 2 without the restriction, configurations with predicted energy uncertainty up to 10 eV/atom are generated. Most of these correspond to configurations containing unphysically small interatomic distances, as also shown in Fig. 4. When the restriction of a 62.5 meV/atom cutoff is applied during sampling, unfamiliar configurations are still sampled, but they remain physical; there are no short interatomic distances or large volumes that would impede a DFT calculation.

**Fig. 5:**
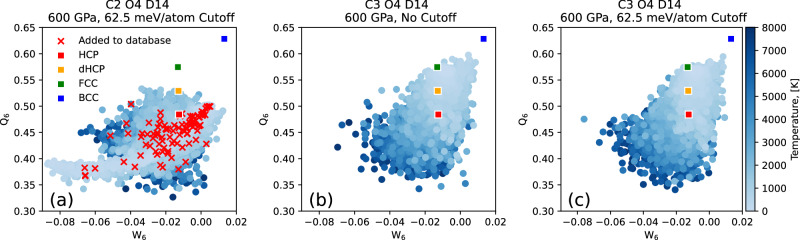
The distribution of *Q*_6_ and *W*_6_ Steinhardt bond order parameters, for the samples at 600 GPa, across cycles, with and without the maximum STD restriction enabled. **a** Shows the distribution from cycle 2 with the restriction enabled, **b** and **c** show the distributions from cycle 3 without and with the restriction enabled, respectively. Samples are coloured by temperature with red crosses indicating the samples added to the database. We highlight the drastic change in distribution from cycle 2 to 3, and the minimal effect of the restriction through the similarity between (**b**) and (**c**).

In cycle 3, NS was run across the entire pressure range of interest again. Since none of the high pressure runs stopped due to the STD stopping criteria, the STD restriction was removed. From these NS runs 10 configurations were selected that were equally spaced in iteration number from the final configuration up to 1000 K. After this cycle, 390 16-atom configurations were evaluated using DFT and added to the database, for a total of 4330 16-atom configurations. To show that the STD restriction is a suitable selective identifier of PES holes, in column **a** of Fig. 4 when PES holes are encountered the STD spikes to around 10 eV/atom but in column **c** when the holes have been fixed the STD only spikes to around 0.1 eV/atom. Additionally, to demonstrate that the restriction does not affect the sampling when MLIP PES holes are not encountered, Fig. 5 shows the distribution of samples in *W*_6_ - *Q*_6_ parameter space when a hole is encountered and no restriction is applied (a), when a hole is not encountered and the restriction is not applied (b), and when a hole is not encountered and the restriction is applied (c). The indistinguishable differences between distributions b and c support the use of this restriction during sampling.

While subsequent NS simulations did not find more unphysical configurations, additional configurations did improve our benchmarks metrics. In this stage of the active learning process we weighted our samples according to Eq. (4), thus lower enthalpy configurations have greater importance. To avoid overweighting the low pressure configurations, the weights were rescaled at each sampled pressure. This weighting scheme also ensures that high-energy and unphysical configurations have lower weights associated with them, as accuracy in the corresponding regions of the PES is less important. We would like to emphasise, that our procedure does not guarantee that all holes are eliminated; while unbiased and exhaustive NS exploration has not identified further unphysical regions, there is a possibility that a higher resolution sampling (i.e. employing more walkers), a larger system size, different thermodynamic conditions, or with a more flexible MLIP model, further PES holes could be sampled.

Cycle 3 has produced an excellent general purpose MLIP, C4 O4 D14, capturing the expected thermodynamic properties of magnesium as shown in detail below. For cycle 4, in order to improve the prediction of low-enthalpy microscopic properties, we performed a fourth cycle of our procedure. For computational efficiency, and to be able to evaluate more samples concentrating on low entropy phases, NS was performed with 8 atoms. The final 100 configurations from each sampled pressure were evaluated with DFT and added to the database. Our final database contains 8230 configurations with 100,480 atoms in total.

### 0 K enthalpy curves and isotropic volume expansion

**Fig. 6:**
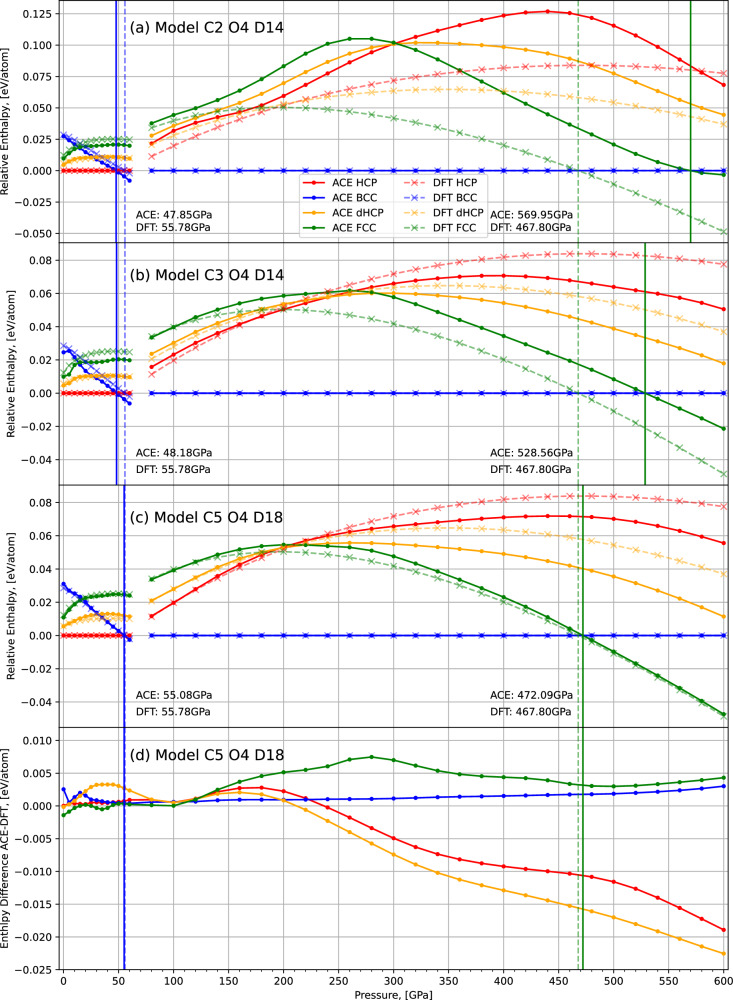
The 0 K enthalpy minima of the four key crystal structures of magnesium, up to 600 GPa, for three different ACE models compared to DFT. Panels **a**–**c** use the ACE models used to generate samples in cycle 2, 3 and 5 respectively. **d** Shows the enthalpy differences between the ACE and DFT predictions in (**c**). Vertical lines indicate a change of the ground state structure. Note that at 60 GPa, due to the change in the ground-state structure, we change from the HCP reference to the BCC reference creating a discontinuity in how the lines are shown.

To benchmark the ability of the MLIP to predict the relative stability of different crystal structures, and thus to identify the ground state, we calculated the enthalpy at 0 K for BCC, FCC, HCP, and dHCP structures. Figure 6 shows these results in the 0–600 GPa pressure range, obtained at different stages of our active learning procedure. We would like to emphasise that none of these crystalline structures have been manually added to the database during the training and while NS performed with the EAM potential in cycle 0 sampled a range of relevant solid structures, these provided limited and often incorrect lattice parameters. Despite this, the C2 O4 D14 potential trained at the end of cycle 1 already provides reasonable predictions of relative phase stabilities (Fig. 6 panel (a)), particularly at low pressures, and while the relative enthalpy difference between DFT and the ACE potential deviates more at extreme pressures, the relative stability order between phases is already correct. This is a testament to both the ability of the ACE architecture to accurately interpolate, based on only a small amount of data, and the quality of data collected through our procedure.

As the high pressure phases are sampled more extensively during cycles 3 and 4, enthalpy predictions improve. However, we found that significant improvements can only be achieved by increasing the flexibility of the model, rather than by additional samples, and thus we increased the degree of our model (C5 O4 D18) – increasing flexibility and computational cost – and refitted to the final database. This new model provides excellent agreement with the HCP to BCC, and BCC to FCC phase transition pressures.

One might also notice that the enthalpy predictions of the high-pressure metastable phases, HCP and dHCP, are less accurate than those involved in the phase transition, FCC and BCC (Fig. 6d). This naturally emerges from our database building procedure, as NS samples the most thermodynamically relevant basins, hence the metastable structures are less well represented in the training data. Selectivity towards thermodynamically relevant basins increases computational efficiency when constructing databases and avoids the evaluation of unimportant configurations.

**Fig. 7:**
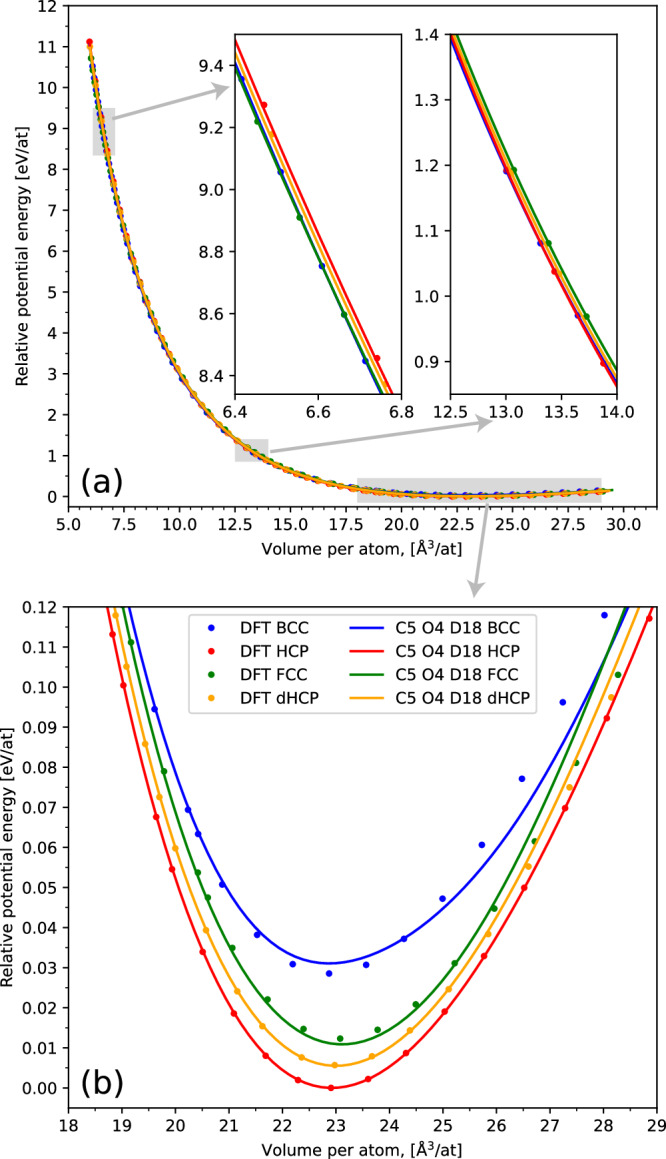
Energy as a function of volume for the four key crystal structures of magnesium, calculated using DFT and our final ACE model, C5 O4 D18. **a** DFT results are shown as single points, while model predictions are shown as solid lines. **b** Shows the minimum energy region of the curves. Note, the cells were expanded isotropically so for HCP and dHCP, other than at 0 GPa, these are not related to the enthalpy minima.

We also present the potential-energy minima isotropic volume expansion curves, shown in Fig. 7, as a demonstration of the functionally smooth local minima, absent of any potential-energy holes up to very small cell volumes. We also compare these results to those obtained via DFT of the same structures and observe excellent agreement for the lowest energy phases on the left-hand side of the graph. As explained above, we do not expect perfect results from phases that are not thermodynamically relevant, such as a high-volume BCC crystal, and since the NS calculations generating the training data were not performed at negative pressure values, it is expected that on the right-hand side of the curve, corresponding to high-volume crystal structures, the predictions will be poor. The results remain physical despite the lack of data.

### BCC-FCC transition pathway (Bain path)

In order to benchmark the behaviour of the potential in regions of phase space that are important for determining the mechanics of phase transitions, we evaluated the transition pathway between BCC-FCC phases, known as the Bain path, shown schematically in Supplementary Fig. 2.

**Fig. 8:**
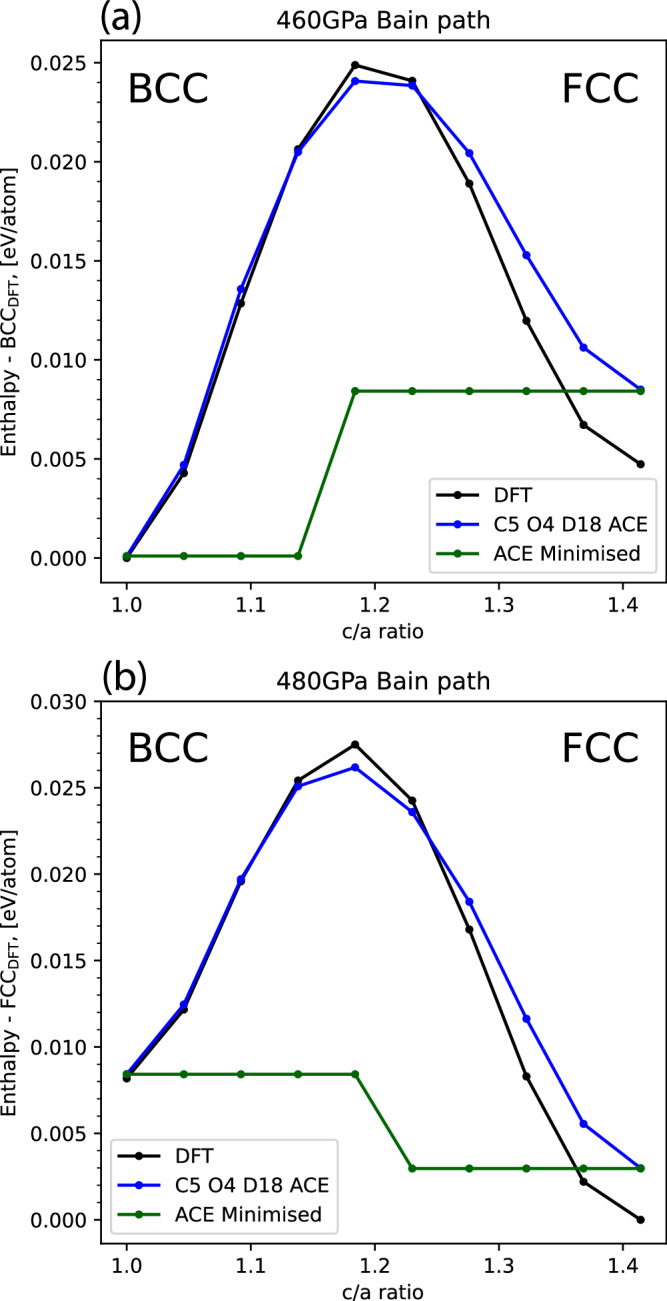
The enthalpies of BCT intermediate structures along the Bain path at different pressures. **a**, **b** Show the results at 460 GPa and 480 GPa respectively. DFT results are shown in black with C5 O4 D18 ACE model predictions in blue. We minimised these intermediate structures using the ACE model, to show no spurious minima along the pathway exist, and plot the enthalpies of these minimised structures in green. The Bain path is shown schematically in Supplementary Fig. 2.

The results in Fig. 8 show the Bain path at two different pressures, one where the BCC phase is the most stable and at higher pressure when FCC is the ground state. These show that the enthalpy of the FCC phase is overestimated, which was also observed from the ground state enthalpy comparison plots (Fig. 6), but the BCC phase is in excellent agreement with the DFT results at both pressures. Due to the slight underestimation of the transition point between the phases and the overestimation of the FCC trough, it seems to suggest the model better fits to the surface at finite temperatures rather than 0 K.

### Phonons

The phonon spectra are representative of how well the MLIP can reproduce the forces within potential energy basins in specific high-symmetry directions. This benchmark can be challenging as it depends on the gradients of the potential energy landscape, which requires dense sampling around the minimum to correctly approximate. Given we do not explicitly supply the basin minima, or direct sampling along high-symmetry directions, this benchmark could be particularly challenging.

**Fig. 9:**
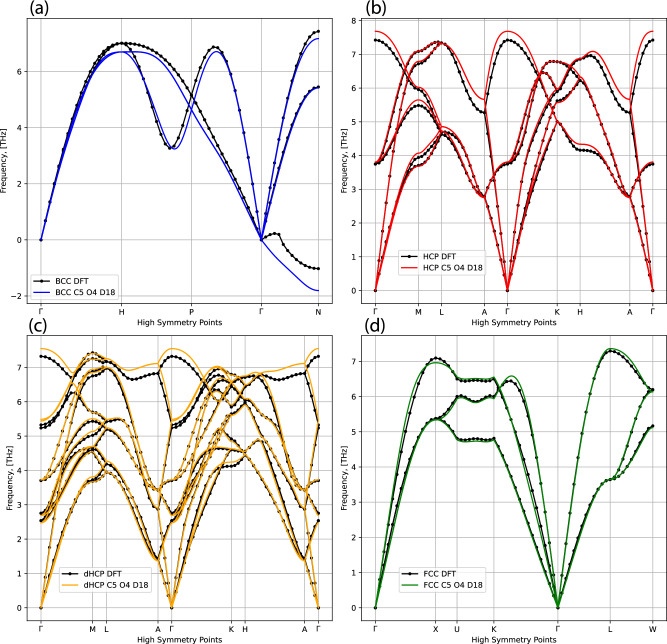
Phonon spectra for the four key crystal structures at 0 GPa. Spectra were calculated using DFT and our final ACE model, C5 O4 D18. DFT results are shown in black, with ACE results given for BCC in blue (**a**), for HCP in red (**b**), dHCP in orange (**c**), and for FCC in green (**d**).

We present the phonon spectra at 0 GPa in Fig. 9, where we demonstrate good agreement with the DFT benchmarks across all four crystal structures identified in the current work. We also detect softening of the unstable BCC phonon mode, even though BCC is not the lowest enthalpy phase at 0 GPa and subsequently not well represented in the database.

**Fig. 10:**
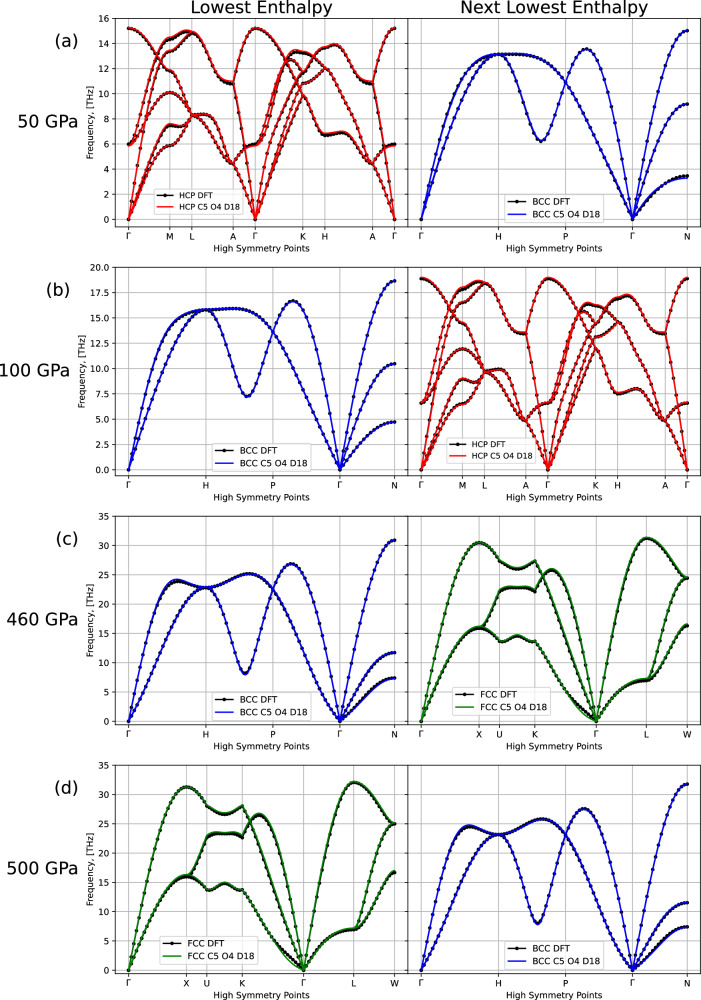
Phonon spectra for FCC, BCC, and HCP, around the 0 K phase transition pressures. Spectra were calculated using DFT and our final ACE model, C5 O4 D18. With the HCP-BCC and BCC-FCC ground state transitions occurring at 56 GPa and 468 GPa, respectively, we compare the HCP (red) and BCC (blue) phonons at 50 GPa (**a**) and 100 GPa (**b**), and the BCC (blue) and FCC (green) phonons at 460 GPa (**c**) and 500 GPa (**d**), to DFT (black).

Compared to previous studies^75^, we are not expecting a uniformly excellent agreement across the phases, as thermodynamically unstable phases are undersampled or not sampled at all in our approach. We demonstrate, however, that phonon dispersions at finite pressures, as presented in Fig. 10, show excellent agreement with the DFT reference for the thermodynamically stable phase and the next lowest enthalpy phase across a broad range of pressure values. This result indicates our procedure is working as expected from the sampling properties of NS and that our MLIP can be trusted to reproduce difficult properties of thematerial under near-equilibrium conditions.

### Elastic constants

The elastic constants provide a measure of the accuracy of the stresses predicted by our MLIP at 0 GPa. We present the elastic constants for the four principal crystal structures of magnesium compared to DFT in Table 2. At 0 GPa the most stable phase is HCP and therefore the most sampled by NS and thus highly accurate, but the elastic constants for the other metastable solid phases also show excellent agreement, with the largest differences only on the order of 5 GPa compared to DFT.

**Table 2:** Key components of the elastic constant matrix of the four key structures, calculated using DFT and our final ACE model, C5 O4 D18

Component	HCP		dHCP		FCC		BCC	
	DFT	ACE	DFT	ACE	DFT	ACE	DFT	ACE
C11	64.61	63.82	63.22	60.25	43.47	47.05	33.54	24.67
C12	22.97	23.07	23.59	24.78	31.11	30.36	35.99	29.61
C13	21.16	20.79	20.69	21.22	–	–	–	–
C33	64.68	68.62	64.59	68.11	–	–	–	–
C44	17.59	19.04	15.39	14.88	23.76	18.49	29.85	31.34

All units are in GPa.

### Phase diagram

**Fig. 11:**
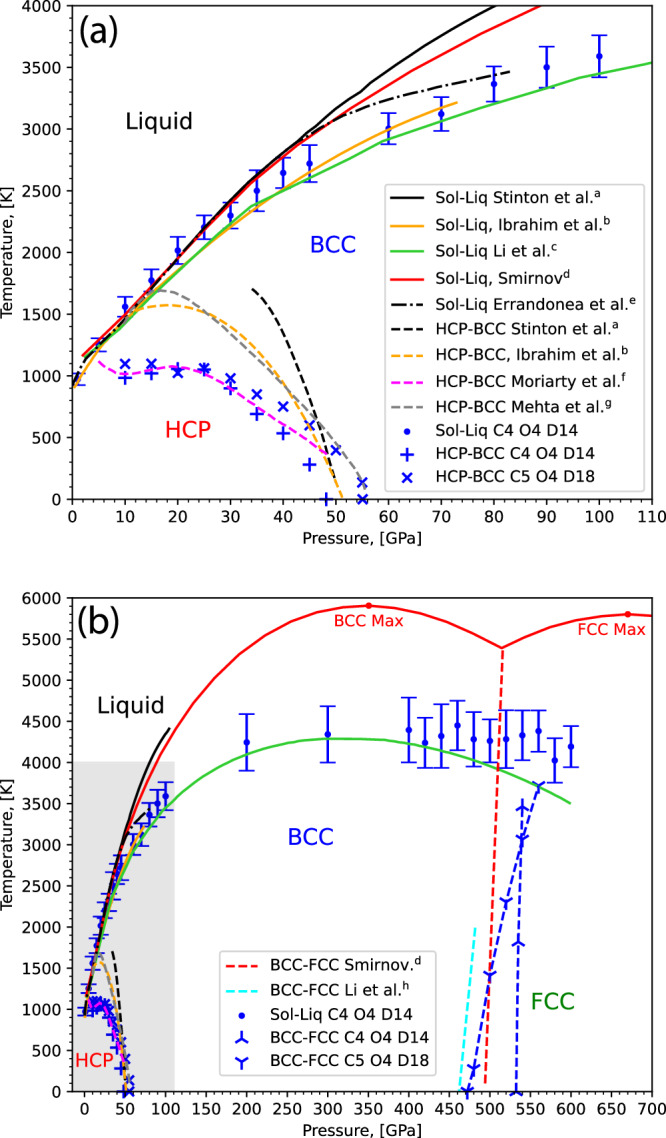
Pressure-temperature phase diagram of magnesium. We compare predictions evaluated using the C4 O4 D14 ACE potential developed in the current work (blue symbols) to previous experimental measurements and computational predictions (a:^66^, b:^75^, c:^74^, d:^73^, e:^64^, f:^67^, g:^68^, h:^71^). The error bars on our NS results represent the full-width half-maximum of the calculated constant pressure heat capacity peaks (discussion on this can be found in Supplementary Section II). Panel **a** shows the lower pressure grey region of the phase diagram in panel (**b**), enlarged.

To comprehensively evaluate the phase behaviour of our ACE model, we calculated the phase diagram across a wide pressure range of 1−600 GPa as shown in Fig. 11. We employed NS to perform an unbiased exploration of the configurational space, ensuring that all relevant phases are considered and that no erroneously stabilised structures influence the results. Through NS our MLIP predicts the experimentally expected phases of magnesium across a very wide pressure range, without failures due to holes in the PES. All results are in close agreement with existing data where available, further validating the predictions made by our model.

First, looking at the melting line between 1 and 45 GPa, there is excellent agreement with both existing experimental data and other computational studies. Beyond 50 GPa, our results, and those of other computational studies, agree best with those reported by Errandonea et al.^64^, in contrast to those reported by Stinton et al.^66^.

At high pressures, where experimental melting data has not been collected, our results agree very closely with recently published ab initio results from Li et al.^74^ for the BCC melting line up to 400 GPa. We note that our predicted melting temperatures are consistently above those of Li et al. but within 200 K. NS results typically suffer from finite size effects that push transition temperature predictions above their ideal limit, so this trend is to be expected, and our results could be further improved by incorporating finite size effect corrections. The error bars correspond to the width of the heat capacity peaks, whose broadening is a direct result of the finite size effect due to the system size of 64 atoms. This is further discussed in Supplementary Section II.

Past 400 GPa as pressures approach the BCC-FCC solid-solid transition, our results begin to deviate from those of Li et al.^74^ – which is expected since Li et al. did not consider the FCC phase in this region. The melting line above 400 GPa becomes relatively flat, similar to what has been predicted computationally both by Li et al. and Smirnov^73^, although the transition temperatures in the latter are considerably higher.

**Fig. 12:**
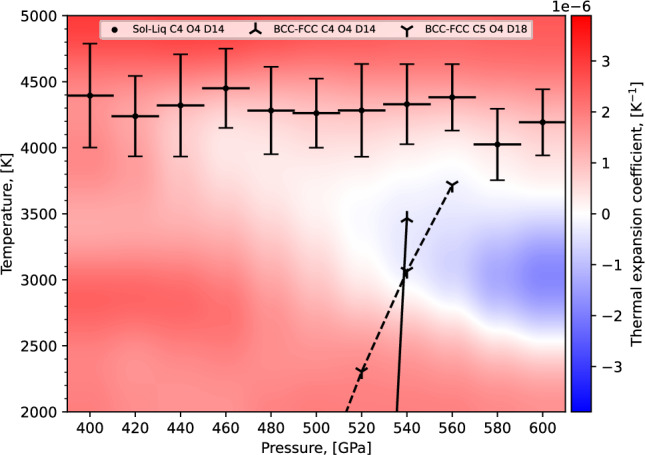
Thermal expansion coefficients between 400 and 600 GPa. Calculated from the NS runs with our C4 O4 D14 ACE model. The melting points are displayed as horizontal black bars and the QHA prediction for the BCC-FCC solid-solid transition is shown with triangular black markers. The red regions show the typical behaviour of volume increasing with increasing temperature, while the expanding and darkening blue region with increasing pressure demonstrate negative thermal expansion. Note to remove some of the noise resulting from differentiation of stochastic averages, we have applied Gaussian filtering with widths of 200 K and 20 GPa on the temperature and pressure axes, respectively. The unfiltered data is provided in Supplementary Section VII.

To gain further insight into the character of the melting line, we determined the thermal expansion coefficients across the NS runs, which is shown in Fig. 12. Across the NS runs from 400 to 440 GPa there is positive thermal expansion up to 460 GPa where the thermal expansion becomes negative indicating a maximum of the BCC melting line within the pressure range of 440–460 GPa. Thermal expansion remains negative, and becomes more so, up to the final measured pressure of 600 GPa.

**Fig. 13:**
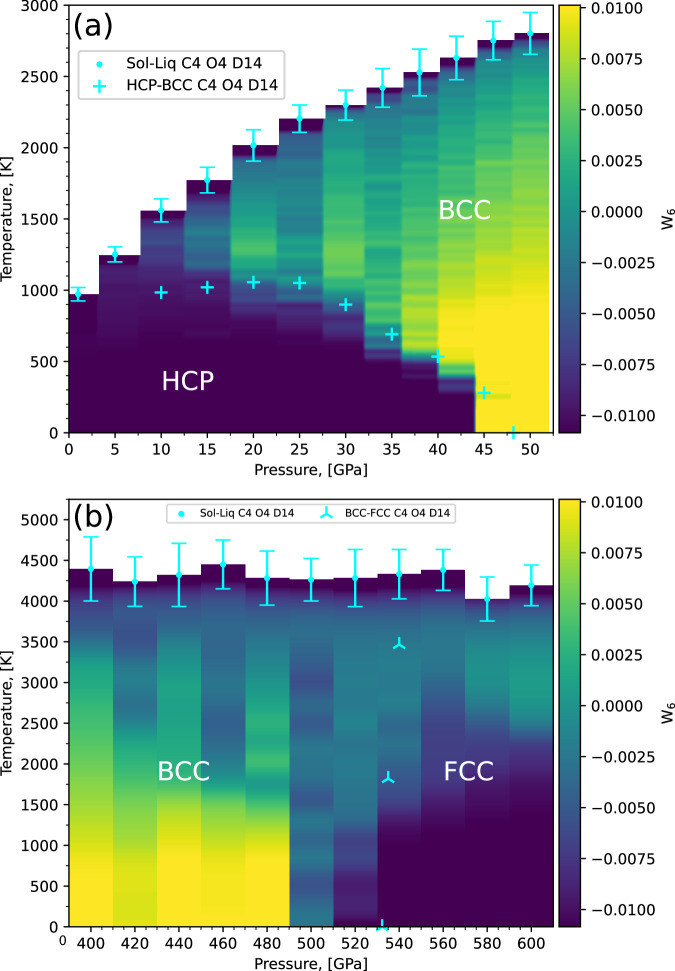
*W*_6_ Steinhardt bond order parameters around predicted solid-solid transitions on the phase diagram. Configurations were generated from NS of the C4 O4 D14 model with 64 atoms. The yellow regions represent BCC structures and the blue regions represent HCP and FCC structures. **a** Shows the region around the HCP-BCC transition and **b** shows the region around the BCC-FCC transition. Also shown are the predicted melting points (**a** and **b** cyan dots), the QHA for the HCP-BCC transition (**a** cyan plus symbols) and the BCC-FCC transition (**b** cyan triangles). Shown are clear solid-solid transitions, reflecting the expected boundaries. Note the pressure range between 34 and 50 GPa was sampled using replica-exchange-NS^83^.

Due to the difficulty of resolving almost vertical solid-solid transition lines in NS, we used the Quasi-Harmonic Approximation (QHA) to estimate the HCP-BCC and BCC-FCC phase boundaries. Due to the low computational requirements of the QHA, we fitted a higher order potential, C5 O4 D18, after performing an additional cycle and display these results as well. Additionally, while the heat capacity peak is too shallow to pinpoint the exact temperature locations of any solid-solid transitions, they are clearly observed when looking at the order parameters shown in Fig. 13.

NS correctly samples the expected crystal structures across the entire pressure range, correctly predicting the transition from HCP to BCC to FCC, without being explicitly provided with these structures, and without any external bias. No prior assumptions on the phases were applied in constructing our database or producing the validation results. The QHA for the HCP-BCC solid-solid transition agrees well with DFT based QHA results from Moriarty et al.^67^ and, as discussed by Moriarty et al., the disagreement with experiment stems from entropic effects stabilising BCC at high temperature due to the soft phonon mode seen in Fig. 9. At low temperature stabilisation is caused by electronic effects aligning well with the QHA. Though we are not able to fully reproduce the experimental data, we attribute this to the choice of technique rather than limitations of the model.

For the BCC-FCC solid-solid transition line our results show excellent agreement with the ab initio QHA results produced by Li et al.^71^ and they agree similarly well to that of Smirnov^73^. The boundary is predicted to have a positive, albeit very steep, gradient, but due to the sensitivity of this phase boundary, the gradient value has a high uncertainty, including the possibility of taking negative values.

To complement the thermodynamic information with structural insight, we calculated the average Steinhardt *W*_6_ parameter from our NS simulations. Heatmaps of the *W*_6_ order parameter shown in Fig. 13, indicate a triple point between 480 and 500 GPa, hence there appears to be no inflection point at the triple point like the one suggested by Smirnov^73^ or seen in lithium^86^, and this BCC-FCC transition more closely resembles the character of that seen in sodium and potassium^87–89^.

The position of transitions obtained from the QHA align well with the *W*_6_ order parameter plots but the fact that the 0 K enthalpy transition is at a higher pressure than the NS 0 K enthalpy transition suggests the gradient of the line is negative, contrary to the QHA. Considering the steepness of the gradient of this boundary this prediction is within the uncertainty limitation of our approach, though further finite temperature studies are needed to confirm the nature of the boundary.

### Defects

Through the investigation of defects in magnesium, we show that our database produced automatically from thermodynamic equilibrium structures, is extensive enough to accurately model vacancy formation enthalpies, self-interstitial formation enthalpies, and stacking faults, across our sampled pressure range. In doing so, we demonstrate our MLIP can make valuable predictions under non-equilibrium conditions, even though defect configurations were not explicitly included in the training data.

The vacancy formation enthalpies for HCP, BCC, and FCC under 0, 100, 460, and 500 GPa are given in Table 3 along with the minimum interatomic distance in the relaxed defect structures. Across the broad pressure range of 0–500 GPa, and across the three structures, the largest disagreements between our C5 O4 D18 model and DFT are around 0.2 eV and 0.02 Å.

**Table 3:** Vacancy formation enthalpies, *H*_*vac*_, and minimum interatomic distances, *r*_*min*_, for HCP, BCC, and FCC, at 0, 100, 460, and 500 GPa

Structure	Model	0 GPa		100 GPa		460 GPa		500 GPa	
		*H*_*vac*_	*r*_*min*_	*H*_*vac*_	*r*_*min*_	*H*_*vac*_	*r*_*min*_	*H*_*vac*_	*r*_*min*_
HCP (47 atoms)	DFT	0.79	3.15	4.69	2.42	–	–	–	–
	C5 O4 D18	0.67	3.14	4.81	2.42	–	–	–	–
BCC (35 atoms)	DFT	0.49	3.05	3.95	2.40	8.41	1.98	8.22	1.89
	C5 O4 D18	0.35	3.03	4.14	2.40	8.15	1.99	8.13	1.88
FCC (35 atoms)	DFT	–	–	–	–	9.10	1.95	9.41	1.92
	C5 O4 D18	–	–	–	–	8.86	1.96	9.25	1.93

Values were calculated using DFT and using our C5 O4 D18 ACE model. *H*_*vac*_ is in eV and *r*_*min*_ is in Å.

In Table 4 we present the stacking fault formation energies, under 0 GPa, for four key structures that we identified in the X-Ray Diffraction Patterns Section. As above, these non-equilibrium formation energies show excellent agreement with DFT, with disagreements on the order of 0.005 eV.

**Table 4:** Stacking fault formation energies from HCP at 0 GPa for four key stacking variants

Structure	Formation energy, [eV]		
	DFT	C5 O4 D18	ACE-DFT
ABAC ABAB ABAB	0.022	0.020	−0.00165
ABAC BCBC BCAB	0.036	0.035	−0.00083
ABAC BCBC BCBC	0.042	0.037	−0.00423
ABAC ACAC BCAB	0.042	0.037	−0.00402

Results were calculated using DFT and our C5 O4 D18 ACE model. We found these four structures to express the unassigned XRD peaks in Fig. 15. We also provide the difference in predicted potential energies for the structures to highlight the close agreement.

High energy non-equilibrium properties are typically the most challenging to compute with MLIPs on account of small interatomic distances which often easily leads into PES holes. However, in Table 5 we again show excellent agreement with DFT across the wide pressure range and across the relevant structures. The general trends, such as the increasing enthalpy with increasing pressure, and the decrease between 460 and 500 GPa for the BCC structures, are in complete agreement with the DFT results. Additionally, the difference in interstitial enthalpy predictions varies from around 0.04 eV for the 0 GPa predictions up to around 0.5 eV for the highest pressure enthalpies, which is an excellent agreement across such a broad pressure range. The minimum interatomic distances are also almost exact with the largest differences on the order of 0.01 Å. The exception is the low pressure BCC tetrahedral interstitial defect, due to BCC being unstable at low pressures and therefore poorly sampled, the interstitial interatomic difference is 0.07 Å.

**Table 5:** Self-interstitial enthalpies, *H*_*si*_, and minimum interatomic distances, *r*_*min*_, for HCP and BCC at 0, 100, 460, and 500 GPa

Structure	Site	Model	0 GPa		100 GPa		460 GPa		500 GPa	
			*H*_*si*_	*r*_*min*_	*H*_*si*_	*r*_*min*_	*H*_*si*_	*r*_*min*_	*H*_*si*_	*r*_*min*_
HCP (37 atoms)	B_*O*_	DFT	2.89	2.47	6.66	1.92	–	–	–	–
		C5 O4 D18	2.94	2.47	6.50	1.92	–	–	–	–
	B_*T*_	DFT	3.34	2.54	7.68	1.95	–	–	–	–
		C5 O4 D18	3.38	2.53	7.50	1.95	–	–	–	–
BCC (55 atoms)	*O*	DFT	2.48	2.58	6.06	1.98	6.09	1.67	5.88	1.66
		C5 O4 D18	2.42	2.58	5.96	1.98	6.54	1.67	6.38	1.66
	*T*	DFT	2.00	2.72	5.48	2.08	5.77	1.74	5.59	1.72
		C5 O4 D18	1.93	2.65	5.38	2.08	6.07	1.75	5.91	1.73

For HCP the interstitial atom was placed in the tetrahedral and octahedral sites below the basal plane (B_*T*_ and B_*O*_, respectively), and for BCC, in the tetrahedral and octahedral sites (*T* and *O*, respectively). Values were calculated using DFT and our C5 O4 D18 ACE model. *H*_*si*_ is in eV and *r*_*min*_ is in Å.

We find these benchmarks remarkable since our database consists of only samples automatically produced as a function of thermodynamic relevance.

### X-Ray diffraction patterns

**Fig. 14:**
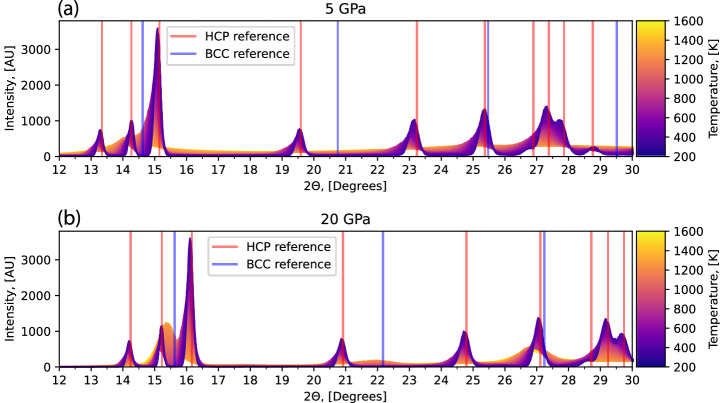
XRD powder spectra calculated at different temperatures and pressures. Using the XRD patterns of the samples collected from sampling the C4 O4 D14 model with 64 atoms at 5 GPa (panel **a**) and 20 GPa (panel **b**), we show the XRD patterns (coloured by temperature), against peaks for the enthalpy minimised BCC structure (blue) and HCP structure (red).

The final property we investigated was the temperature dependent X-Ray Diffraction (XRD) patterns, in order to interpret the additional peaks recorded by Stinton et al. at a 2*θ* angle of 15.9^∘^ and 25.6^∘^^66^. We did not observe any indication of these peaks, indicating that it is unlikely that these peaks correspond to an unidentified stable phase. It follows that if they correspond to a single crystalline phase, it could only correspond to a metastable form. In Fig. 14 we present the temperature dependent XRD patterns at 5 and 20 GPa to show that only the HCP and BCC phases are sampled in a substantial amount.

**Fig. 15:**
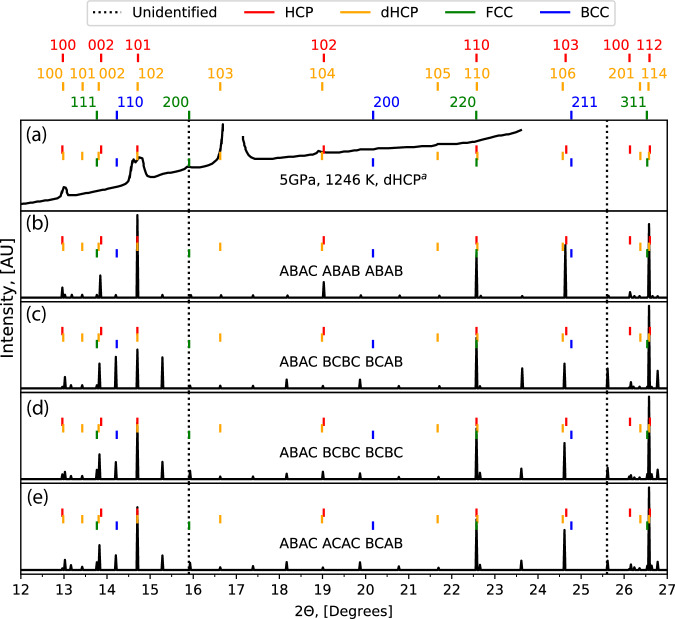
Comparison of calculated XRD patters to experimental measurements. We present the XRD patterns for four 12-layered close-packed structures (panels **b**–**e**), which all have the experimentally observed unassigned peaks, at a 2*θ* of 15.9^∘^ and 25.6^∘^ (black dotted lines), reported by Stinton et al.^66^ (panel **a**). Reference peaks are given from the enthalpy minimised HCP (red), dHCP (orange), FCC (green), and BCC (blue) structures, with the peak labels given in the top row.

While studying the configurations sampled at 1 GPa and high temperatures, we observe a range of close-packed polytype structures. These polytype structures included amounts of dHCP (ABAC) and FCC (ABC). Further NS runs with 21 atoms allowed us to also sample the 9R structure (ABACACBCB). While none of these structures are predicted to have an XRD peak at 25.6^∘^, FCC shows a peak at 15.9^∘^. Based on this observation, we systematically generated close-packed structures with up to 12 layers to identify if a long-period stacking order was responsible for the unidentified peaks. We found multiple 12 layer structures, shown in Fig. 15, which show peaks at all positions reported experimentally.

### Computational expense

It is important to acknowledge the computational cost of carrying out our suggested procedure, as it has significant implications in terms of energy use and carbon footprint of high-performance computing^90^. We discuss this cost in terms of CPUhrs, where 1 CPUhr is running 1 core for 1 h. We have neglected the computational cost of building the databases and training the linear ACE models as these are negligible compared to the DFT and NS simulations. The cost of the DFT per typical atomic configurations is primarily determined by the composition of the targeted system and would be similar in other comparable MLIP workflows. In general, we aimed for a convergence resulting in sub meV/atom accuracy in our DFT calculations guiding our choice of parameters. All calculations were performed on nodes of 2 AMD EPYC 7742 (Rome) 2.25 GHz 64-core processors. Performing 16-atom DFT with our chosen parameters resulted in a cost of 70 CPUhrs/configuration and with 4330 16-atom configurations in total, the cost amounted to 303.1K CPUhrs. Performing 8-atom DFT with our parameters resulted in a cost of 13.5 CPUhrs/configuration and for 3900 configurations this amounts to a total of 52.7K CPUhrs. In total, around 346K CPUhrs of computational time were used for the DFT calculations of our training data points.

For the NS component, the cost depends on the number of walkers, the walk length, and the model. In turn the model determines the cost with regard to the number of atoms and the pressure. Within the ACE framework, the spatial cut-off of the MLIP is fixed so at higher pressures, due to the decreased volume per atom, there is a significant increase in the number of atoms that fall within the cut-off of the potential, increasing the length of neighbour lists and evaluation times, leading to a marked rise in computational expense. Table 6 shows the cheapest and most expensive NS runs with varying the model, the number of atoms, the number of walkers, and the pressure.

**Table 6:** CPUhrs taken for NS with different numbers of atoms at different pressures

Potential	Atoms	Pressure	Cost	Wall time
		[GPa]	[CPUhrs]	[hrs]
EAM^a^	16	0	408	8.5
ACE	16	0	252	9.0
ACE	64	1	3144	131.0
ACE	16	600	1120	46.0
ACE	64	600	11,648	485.0

The walk length was the same in all cases.

^a^This was performed with four times as many walkers as with the ACE.

### Summary

In this study, we proposed and demonstrated a new framework for generating training data to develop MLIPs. Our approach stands out in its ability to automate the construction of a database based on thermodynamically relevant configurations rather than relying on human-driven selection of structures or pre-existing knowledge of certain phases. This ensures that the resulting MLIP captures phase properties reliably across a wide range of conditions.

Our procedure adapts an iterative training cycle to improve the performance of the MLIP model, with the NS technique being at the heart of each cycle due to its ability to explore the relevant configuration space in an unbiased way. NS provides both critical information about the performance of the model and generates important configurations that need to be incorporated into the training dataset. This is particularly important for creating reliable MLIPs that are robust across volume, pressure, and temperature variables.

By applying this framework to magnesium as a test case, we successfully trained an MLIP using the ACE architecture, leveraging the committee STD of total energy predictions, to describe the material’s behaviour over an extensive pressure-temperature range, covering liquid and solid phases up to 600 GPa. We used an EAM model as a starting point, and the final potential was achieved after five training cycles, with the final database consisting of 4330 16-atom configurations and 3900 8-atom configurations. We constructed two ACE models with: body order 4, degree 14 and a total of 710 basis functions; and a higher accuracy model with body order 4, degree 18 and a total of 2849 basis functions. Both potentials demonstrated excellent agreement with benchmark calculations, including geometry optimizations, phonon spectra, elastic constants, vacancy and self-interstitial formation enthalpies, and the phase diagram.

We used our potentials to probe the phase diagram at pressures which are challenging to achieve in experiments and to explore configuration space where experimental results are not completely explained.

In conclusion, the proposed framework represents a powerful and generalisable tool for developing MLIPs, with applications extending to a wide variety of materials and conditions. Its automated, thermodynamically informed, and extensible nature makes it a significant step toward overcoming current challenges in the field and enabling more accurate and efficient materials modelling.

## Methods

### Nested sampling

All NS calculations were carried out using the pymatnest software package^91,92^. When generating and expanding the database, some of the NS parameters were changed between active learning cycles. At each cycle the following parameters were kept constant. NS was performed starting from a maximum volume of 500 Å^3^/atom and progressing the sampling down to 200 K, culling one walker per iteration. The walk length for each walker retained the same ratio of moves given in Table 7. A minimum allowed cell aspect ratio of 0.65 was used which was increased to 0.95 for the 64-atom NS^93^. When we restricted accepted walk moves to produce configurations where the STD of total energy predictions from the committee are below 62.5 meV/atom, we stopped the NS run if 90% of the walkers had a value above 60.625 meV/atom.

**Table 7:** MC moves used during NS

Move type	Proportion	Acceptance rate
5-step TEHMC^a^	0.21	50–95%
Volume Change	0.31	25–75%
Cell Shear	0.24	25–75%
Cell Stretch	0.24	25–75%

Provided are the type and proportion of different MC moves used during NS walks and the acceptance rates used to dynamically adjust step sizes.

^a^Total enthalpy Hamiltonian Monte Carlo^91^.

Across the cycles, the number of walkers, *K*, the sampled pressures, the walk length, and maximum committee STD was varied due to the associated cost, required accuracy, and stability of the different models, as discussed in detail in the previous sections. These parameters are given in Table 8.

**Table 8:** NS parameters used across the active learning cycles

Cycle	Atoms	Pressure range	*K*	*L*	Comm. STD
		[GPa]			[meV/atom]
0	16	0, 1, [5–45,5]	1248	1248	N/A
1	16	0, 1, [5–45,5], [60–600,20]	336	336	62.5
2	16	0, 1, [5–45,5], [60–600,20]	336	336	62.5
3	16	0, 1, [5–45,5], [60–600,20]	336	336	None
4	64	0, 1, [5–45,5], 100,200,300	336	336	62.5
4	64	[60–90,10], [400–600,20]	384	336	None

The number of atoms, number of walkers, *K*, number of proposed steps in decorrelating the configurations between iterations, *L*, and the maximum accepted STD of energy predictions made by the committee of models (meV/atom).

### ACE models

All ACE fitting was done using the ACEsuit Julia software package^13,94^. The cutoff for constructing the ACE was set to 8.2 Å, as at this distance the DFT pair potential is less than 10^−8^ eV/atom.This value has also been used in related studies of magnesium^75^. A single atom reference energy of −1688.821 eV was used, calculated by placing a single Mg atom in a suitably large cubic cell.

In order to pick ideal parameters for the ACE model, once the initial database was constructed, 20% of the configurations were randomly selected and removed from the training set, forming a test set. The model was then trained on the remaining 80% of the data and the accuracy of the fit was determined by calculating the Root Mean Square Error (RMSE) of the predicted energies of the test set. During the initial fitting, the configurations were weighted equally, and the energies, forces, and virial stress components were weighted with a ratio of 9:1:1. The chosen body order was set at 4 and the degree was set at 14, for a potential consisting of 710 basis functions. Once these parameters were determined, a refit was performed using the full database. The RMSE for the fit of this potential to the training data was 1.3 meV/atom, 19 meV/Å, 10.7 meV/atom, for the total energy, forces, and virials respectively. Bayesian linear regression was employed for the fitting and from the produced posterior distribution, ten parameter sets were drawn, forming a committee of potentials^95^. This was used to evaluate the uncertainty associated with energy predictions made by the ACE model, by calculating the STD of the ten total-energy predictions made by the committee.

In our active learning procedure, we used Eq. (4) to calculate the individual weight, *W*_*i*_, for each configuration in the loss function, using the enthalpy difference between the configuration generated at the *i*-th iteration, *H*_*i*_, and the enthalpy of the final sample, *H*_*F*_ generated during a NS run. We controlled this exponential through parameter *α* and found a value of 0.1 minimised the RMSE during fitting.

4 $${W}_{i}={e}^{-\alpha ({H}_{i}-{H}_{F})}$$

### DFT

All configurations within the database were evaluated with the CASTEP DFT software package^96^, using the Perdew–Burke–Ernzerhof (PBE) exchange correlation functional^97^. Mg was represented by an on-the-fly generated ultra-soft pseudopotential based on the C19 definition in CASTEP, with a core radius of 1.8 Bohr and 10 valence electrons explicitly considered in the configuration [2s2 2p6, 3s2]. A plane wave cutoff of 700 eV was used with a fine grid scale of 4.0 and an Self-Consistent Field (SCF) convergence tolerance of 10^−9^ eV. Monkhorst–Pack (MP) k-point grids, with a maximal grid spacing of 0.015 Å^−1^, were used to sample the Brillouin zone and we applied Gaussian smearing to the occupancies with a width of 0.2 eV to improve convergence. Convergence tests and support for these DFT parameters can be found in Supplementary Section V.

### Phonons

The DFT phonon spectra were calculated using the finite displacement method implemented in the CASTEP software package utilising non-diagonal supercells^96,98^. In addition to the parameters specified in the DFT Section, a finite displacement of 0.05 Å was used on minimum enthalpy structures produced using the parameters specified in the Enthalpy Minimisations and Defects Section. A q-grid of 4 × 4 × 4 was used and interpolated to a finer grid with maximal grid spacing of 0.1 Å^−1^ along the high-symmetry paths to produce the DFT phonon spectra.

The ACE phonon spectra were calculated using the phonopy Python software package^99,100^. Supercells of size 4 × 4 × 4 were constructed for the four key crystal structures (HCP, dHCP, FCC, and BCC) and finite displacements of 0.05 Å were used to determine the force constant matrices.

### Elastic constants

Both the ACE and DFT elastic constants were calculated using the matscipy Python software package^101^. In both cases the unit cells were first relaxed, and the finite strains were applied in increments of 5 × 10^−5^. The increments were chosen such that decreasing the finite strains further resulted in no significant change in the elastic constants. The parameters given in the DFT Section were used, except all k-point grids were fixed to those in Table 9.

**Table 9:** Fixed MP k-point grids used for elastic constant and enthalpy minimisation calculations for the different crystal structures unit cells

Crystal structure	MP k-point grid
HCP	38 × 38 × 20
dHCP	38 × 38 × 10
BCC	42 × 42 × 42
FCC	41 × 41 × 41

These were chosen as these are the grids generated from a 0.015 Å^−1^ maximum grid spacing for the minimum enthalpy unit cell structures at 600 GPa.

### Enthalpy minimisations and defects

All DFT calculations were performed using CASTEP with the parameters specified in the DFT Section, unless otherwise specified below^96^. Tolerances of 0.02 meV, 1 meV/Å, 0.01 GPa, and 0.001 Å were used to determine convergence for the total energy, forces, stresses, and atomic displacements, respectively, for any DFT geometry optimisations. Equivalent calculations using a MLIP were performed using the Atomic Simulation Enviornment (ASE) software package, with a force tolerance of 10^−5^ eV/Å to determine convergence^102^.

For calculating the enthalpy minima, the symmetry of the unit cells was maintained by fixing the angles and ratios of relevant cell parameters during optimisation, and to avoid k-point re-meshing, fixed k-point grids, given in Table 9, were used throughout the minimisations.

To calculate the vacancy formation enthalpies, we used the minimum enthalpy structures under the pressures specified in Table 3, to construct supercells of HCP, BCC, and FCC, and a single atom removed. With the lattice parameters fixed, the structures were relaxed to the tolerances specified above. The formation enthalpy was then calculated using Eq. (5).

5 $$\begin{array}{lll}{H}_{vac}\,=\,\left({U}_{vac}(N-1)-\frac{N-1}{N}{U}_{bulk}(N)\right)\\\qquad\qquad\,\,+P\left({V}_{vac}(N-1)-\frac{N-1}{N}{V}_{bulk}(N)\right)\end{array}$$

To calculate the self interstitial enthalpies, we used the minimum enthalpy structures under the given pressures to construct supercells of HCP and BCC. For HCP a single atom was added to the *B*_*O*_ and *B*_*T*_ positions,and for BCC a single atom was added to the *O* and *T* sites. With the lattice parameters fixed, the structures were relaxed, and the self-interstitial enthalpy was calculated using Eq. (6).

6 $$\begin{array}{ll}{H}_{si}\,=\,\left({U}_{si}(N+1)-\frac{N+1}{N}{U}_{bulk}(N)\right)\\\qquad\qquad\,\,+P\left({V}_{si}(N-1)-\frac{N+1}{N}{V}_{bulk}(N)\right)\end{array}$$

Finally to calculate the stacking fault formation energies, we used the lattice parameters from the 0 GPa minimum enthalpy HCP structure to construct the four stacking variants provided in Table 4. With the lattice parameters fixed, the structures were relaxed, and the formation energies calculated by subtracting the potential energy of an equally sized pure HCP bulk.

### Bain path

Starting from the BCC unit cell enthalpy minimum at 460 GPa, the unit cell was elongated in the *c* direction and then relaxed with a fixed *c*/*a* ratio at 460 GPa. This was done for ten *c*/*a* ratios from 1 to $\sqrt{2}$ to give the lowest enthalpy pathway along the Bain path. This was done using DFT and the parameters provided in the DFT Section with CASTEP, and with our ACE potential using ASE.

### Stacking variant investigation

To generate the stacking variants systematically, 1–12 atoms were equally placed along *c* in a hexagonal cell (*a*,*a*,*c*, 90^∘^,90^∘^,120^∘^), with fractional *x*-*y* positions of (0,0), (2/3,1/3), or (1/3,2/3). This results in 797,160 possible structures, without correcting for symmetry. After removing equivalent structures, there are 11,676 unique stacking variants, with the quantity of the different layered structures given in Table 10. These configurations were relaxed under 1 GPa and the XRD patterns calculated for a simulated x-ray wavelength of 0.62 Å, using the QUIP software package^103^.

**Table 10:** Number of possible unique stacking variants

Layers	Unique stacking variants
1	1
2	1
3	2
4	4
5	8
6	22
7	52
8	140
9	366
10	992
11	2684
12	7404

After accounting for symmetry, the number of unique stacking variants with up to 12 layers of one species in a hexagonal cell is 11,676, the quantity of unique structures for each *N* layered structure is provided.

## Supplementary information

Supplementary Information

## Data Availability

The data supporting this study’s findings can be found on GitHub at https://github.com/VGFletcher/Fletcher_2025_magnesium_data.

## Code availability

The open-source package pymatnest is freely available on GitHub at https://github.com/libAtoms/pymatnest. Python functions to construct databases from pymatnest output files can be found at https://github.com/VGFletcher/NS_database_builder.
