# Supplementary material for: Autonomous thermodynamically informed database generation for machine-learned interatomic potentials and application to magnesium
Source: NPJ Comput Mater. 2025 Dec 17;12(1):36. doi: 10.1038/s41524-025-01903-z (PMC12815665; doi:10.1038/s41524-025-01903-z)
Supplement: Supplementary file 1 — Supplementary Information [file 41524_2025_1903_MOESM1_ESM.pdf]

Supplementary Information for "Autonomous thermodynamically informed database generation for machine-learned interatomic potentials and application to magnesium"

Vincent G. Fletcher,[1,*] Albert P. Bartók,[1,2,†] and Livia B. Pártay[3,‡]

[1] *Department of Physics, University of Warwick, Coventry, CV4 7AL, UK*
[2] *Warwick Centre for Predictive Modelling, School of Engineering, University of Warwick, Coventry, CV4 7AL, UK*
[3] *Department of Chemistry, University of Warwick, Coventry, CV4 7AL, UK*

(Dated: November 4, 2025)

UK Ministry of Defence © Crown Owned Copyright 2025/AWE

I. EAM PHASE DIAGRAM

The phase diagram of the magnesium Embedded Atom Method (EAM) potential, produced by Wilson *et al.*,[1] disagrees considerably with the *ab initio* predictions and experimental observations, as shown in Supplementary Figure 1. While the melting temperatures from 0-15 GPa agree reasonably with the expected results, there is an incorrect face-centred cubic (FCC) phase and a hexagonal close-packed (HCP) to FCC solid-solid transition between 1 and 5 GPa. Additionally, past 40 GPa the correct stable phase of body-centred cubic (BCC) is predicted but the melting temperature is significantly different to that of the expected results and, due to the unrealistic FCC phase, there is an incorrect FCC to BCC solid-solid transition between 35 and 40 GPa. We used this behaviour to produce our initial database.

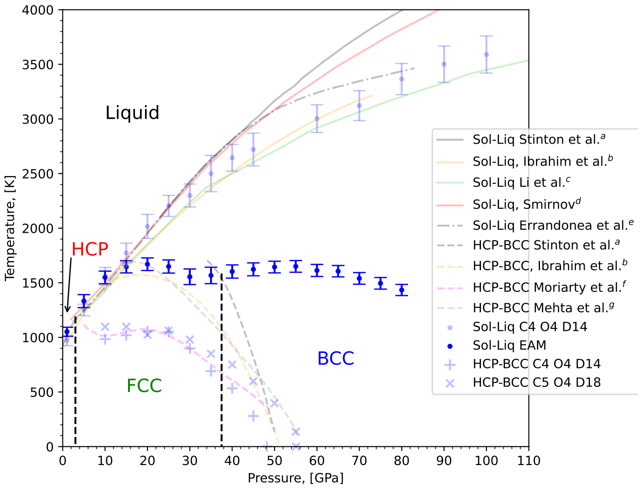

Supplementary Figure 1. **Pressure-temperature phase diagram of magnesium for the Wilson *et al.* EAM model.[1]** Results from the EAM potential are shown in blue, and compared to predictions made using the C4 O4 D14 ACE potential developed in the current work (faded blue symbols) and to previous experimental measurements and computational predictions (a:[2], b:[3], c:[4], d:[5], e:[6], f:[7], g:[8], h:[9]). The error bars on our NS results represent the full-width half-maximum of the calculated constant pressure heat capacity peaks (discussion on this can be found in Supplementary Section II).

II. ERROR BARS IN NESTED SAMPLING

Nested Sampling (NS) is carried out with a finite number of atoms (8-64 in this study), this results in finite size effects that are reflected through a peak on the temperature – heat-capacity plots during phase transitions, rather than a discontinuity that would be seen in the macroscopic system. It is observed that, as system size increases, these peaks become sharper and shift lower in temperature, with the shift becoming increasingly smaller as system size increases.[10] Additionally, it is seen when repeating converged NS runs, the position of the peak, which can shift due to the stochastic nature of the sampling, doesn't shift significantly regardless of system size. Thus, to provide the most meaningful measure of the uncertainty of the position of a phase transition, we provide the Full Width at Half Maximum (FWHM) of the heat capacity peaks taken from a baseline positioned at the tail of the curve which produces the lowest peak prominence.

* Vincent.Fletcher@warwick.ac.uk
† apbartok@gmail.com
‡ Livia.Bartok-Partay@warwick.ac.uk

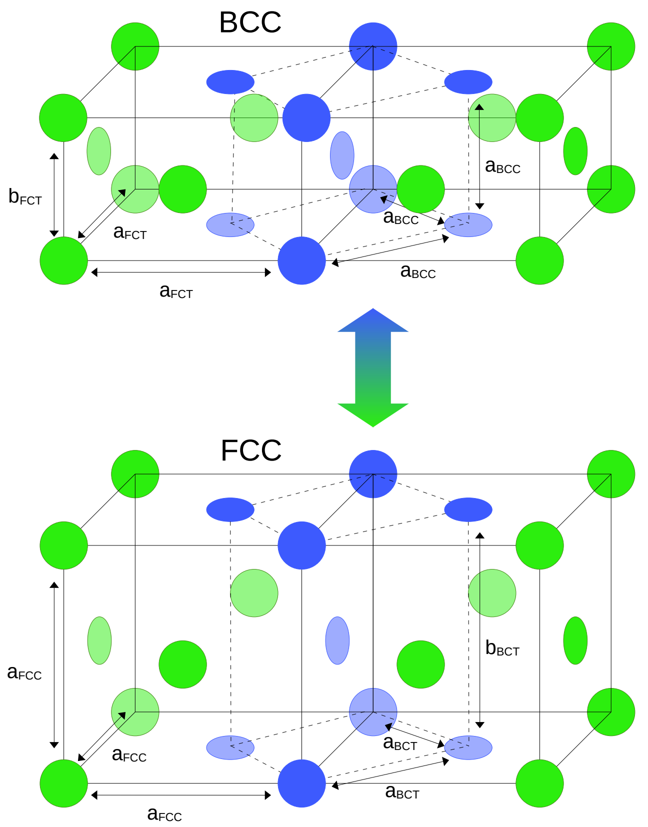

Supplementary Figure 2. **Schematic representation of the Bain transition pathway.** Represented is the transition from BCC to FCC through a BCT transition state, also known as the Bain path.

III. MINIMUM BOND LENGTH RESTRICTION

Before we introduced the use of a committee, to control sampling of Potential Energy Surface (PES) holes, our first solution was to exclude configurations with unphysically short interatomic distances. We were initially concerned with studying magnesium up to 100 GPa, and at this pressure there is a generous interatomic buffer zone to choose a minimum bond length that ensures only the very high-temperature configurations are effected by this restriction. We were willing to allow this, since these configurations are not particularly important for our study. However, when we expanded the pressure range of interest to up to 600 GPa, the parameter choice became difficult to choose such that it did not interfere with the sampling of the liquid phase. Thus we moved away from this solution.

IV. BAIN PATH

A schematic for the lattice deformation from BCC to FCC through a body-centred-tetragonal (BCT) intermediate is displayed in Supplementary Figure 2.

V. DFT CONVERGENCE TESTS

To find converged Density Functional Theory (DFT) parameters, $2 \times 2 \times 2$ supercells of the four unit cells given in Supplementary Table I were constructed. The cell volumes were increased by 5%, the lattice vector components were perturbed randomly by 0-3%, and the atomic positions randomly perturbed by $0 - 0.02$ Å. The DFT parameters were chosen to achieve sub-meV/atom convergence with respect to the total energy, average sub-meV/atom with respect to components of the virial stresses, and average sub-meV/Åconvergence with respect to atomic forces. These results are shown in Supplementary Figure 3.

Supplementary Table I. **Lattice parameters of the initial unit cells used for convergence tests.** Supercells of these unit cells underwent random perturbation, as described in Supplementary Section V, to determine converged DFT parameters.

**Crystal**	**a** (Å)	**b** (Å)	**c** (Å)	α (°)	β (°)	γ (°)	**n atoms**
5 GPa HCP	3.079	3.079	4.99	90	90	120	2
5 GPa BCC	2.983	2.983	2.983	109.47	109.47	109.47	1
600 GPa HCP	2.029	2.029	3.40	90	90	120	2
600 GPa FCC	2.04	2.04	2.04	60	60	60	1

To verify the effect of our chosen convergence parameters, and show that our k-point convergence is acceptable, we increased the density of the Monkhorst-Pack (MP) k-point grids, to those shown in Supplementary Table II, and recalculated the enthalpy minima. This resulted in a 0.12 GPa decrease in the HCP-BCC 0 K phase transition, and a 0.05 GPa increase in the BCC-FCC transition as shown in Supplementary Figure 4. These small changes support our choice of DFT parameters.

Supplementary Table II. **MP k-point grids used during DFT enthalpy minimisations.** To show the effect on the 0 K phase transitions, we used the given sets of fixed k-point grids, to perform geometry optimisations, and calculate the transition pressures.

	**MP k-point Grid**	
**Crystal**	**Original**	**Finer**
HCP	$38 \times 38 \times 20$	$39 \times 39 \times 21$
dHCP	$38 \times 38 \times 10$	$39 \times 39 \times 11$
BCC	$42 \times 42 \times 42$	$43 \times 43 \times 43$
FCC	$41 \times 41 \times 41$	$42 \times 42 \times 42$

VI. PSEUDOPOTENTIAL DELTA TEST

To verify that the ultrasoft pseudopotential used in our DFT calculations was still accurate at 600 GPa, we calculated the delta gauge specified in the paper by Lejaeghere *et al.* and given in Equation 1.[11]

$$\Delta_i(a,b) = \sqrt{\frac{\int_{0.94V_{0,i}}^{1.06V_{0,i}} (E_{b,i}(V) - E_{a,i}(V))^2 dV}{0.12V_{0,i}}} \quad (1)$$

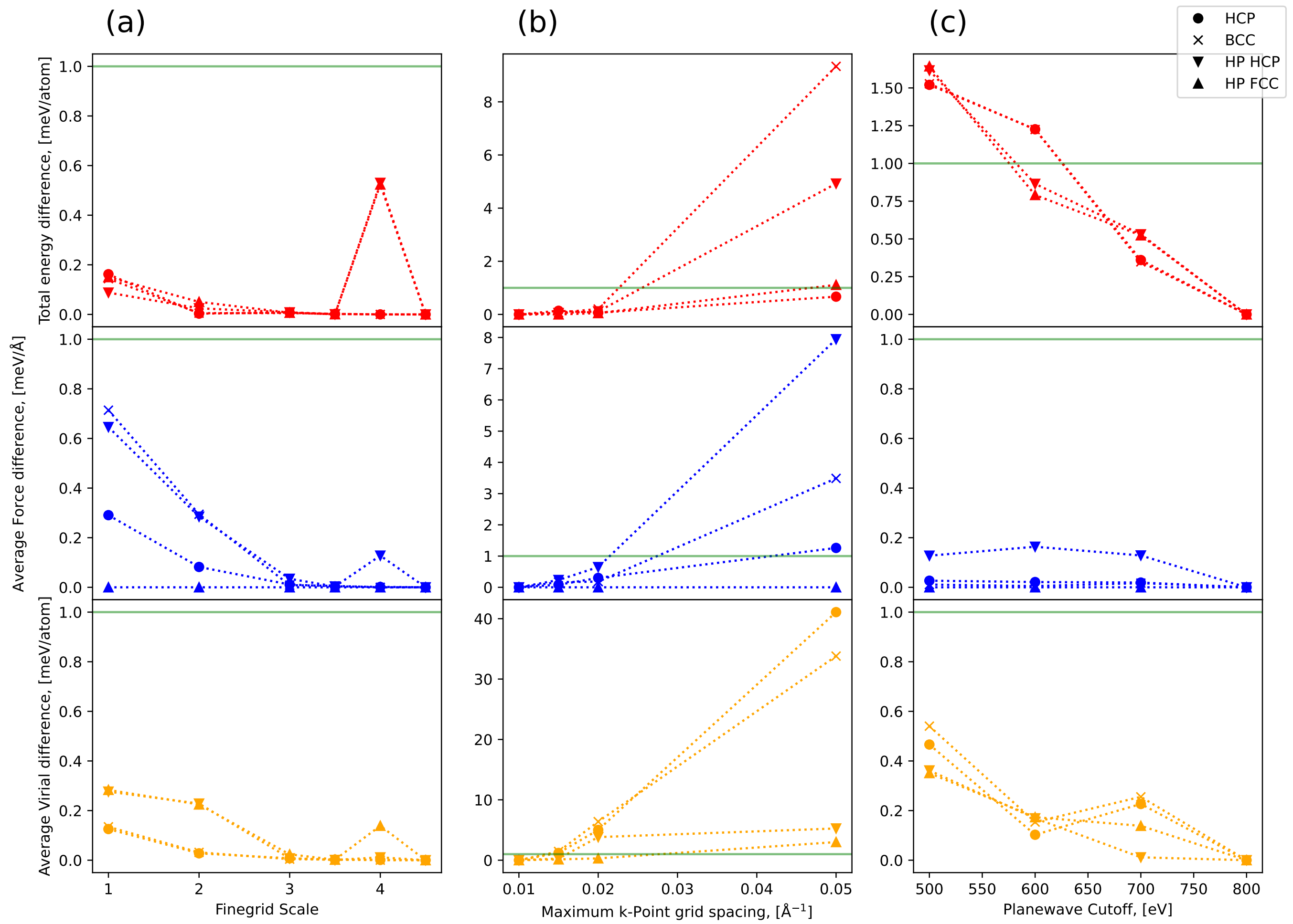

Supplementary Figure 3. **Changes to total energy, forces, and virials, with respect to DFT parameters, for four key crystal structures.** Plots of the difference of total energy (red), the average forces (blue), and average virials (orange) to the most expensive parameter, with respect to finegrid scale (column **a**), maximum k-point grid spacing (column **b**), and planewave cutoff (column **c**). Results are given for supercells of a low and high density HCP structure (dots and down-pointing triangles respectively), a low density BCC structure (crosses), and a high density FCC structure (up-pointing triangles). Horizontal green lines show 1 meV/atom, below which we are satisfied convergence is reached.

To do this, the FCC unit cell - minimised at 600 GPa using the DFT parameters specified in the main paper - was scaled to produce the $E_{b,i}(V)$ curve. Separately, the pseudopotential was changed to the hard pseudopotential specified in CASTEP, and new DFT parameters were determined to achieve sub meV/atom total energy accuracy. This required a plane-wave cutoff of 1200 eV with all other parameters being acceptably converged for the change. The FCC unit cell was minimised again at 600 GPa using the new DFT parameters and this new minimum was scaled to produce the $E_{a,i}(V)$ curve, the key parameter differences are shown in Supplementary Table III. For simplicity, quadratic functions were fitted to the curves to allow easy calculation of the differences and integrals. The measured delta was 0.035 which is acceptably negligible to consider the two calculations in agreement.

Supplementary Table III. **Key parameter changes when moving from the ultrasoft to the hard pseudopotential.** To prove convergence with respect to our choice of ultrasoft pseudopotential, we used a harder construction, and re-converged our DFT parameters. Shown are the differences in the planewave cutoff, needed to achieve convergence, and the change to the FCC lattice parameter.

Property	Ultrasoft	Hard
Plane wave Cutoff, eV	700	1200
FCC a Lat. Par. Å	2.042916	2.041489

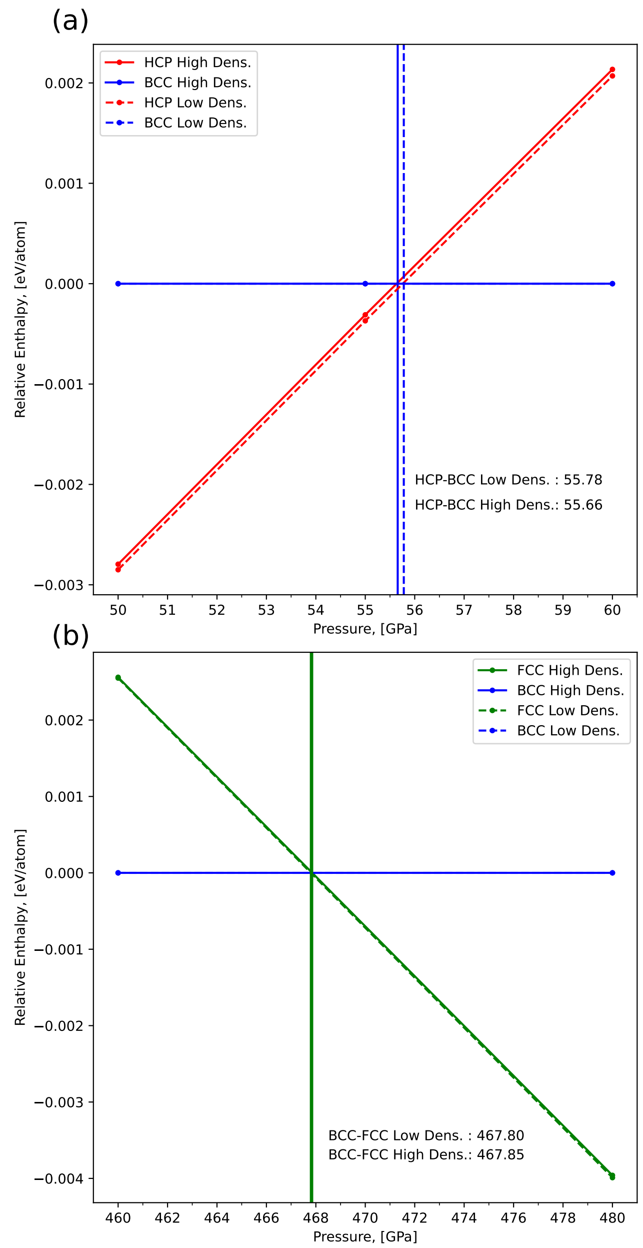

Supplementary Figure 4. **The 0 K transition pressures for the two solid-solid transitions, calculated with different k-point grids. a** shows the HCP-BCC 0 K transition at around 55 GPa, while **b** shows the BCC-FCC 0 K transition at around 467 GPa. Increasing the grid density results in negligible changes to the transition pressures, supporting our choice of grid spacing. The grids are given in Supplementary Table II.

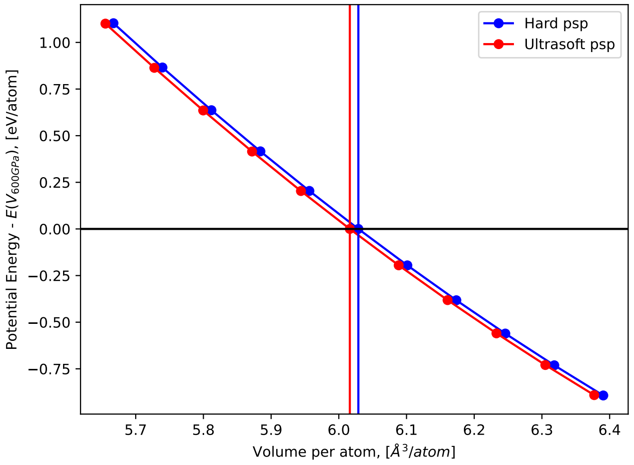

Supplementary Figure 5. **Potential energy from isotropically scaling a unit cell of FCC, from the 600 GPa enthalpy minima of the different pseudopotentials.** Both instances used the enthalpy minimised structure for their respective parameter sets. The close agreement shows a negligible effect of changing to a more accurate pseudopotential at the smallest interatomic distances we investigated.

VII. LATENT HEATS AND ENTHALPY CURVES

With access to the temperature dependent enthalpy curves across the entire pressure range from NS, we can easily compute the latent heats of melting as a function of pressure. Since the temperature dependent enthalpy curves are noisy, we approximate these quantities by fitting a linear function before and after the transition temperature and then calculating the difference between these functions at the transition temperature. This removed some of the noise, however, the temperature range the functions are fitted to affects the latent heat value and so we perform multiple fits, with a buffer range of 100-2000 K, and use the mean and Standard Deviation (STD) of values to provide error bars to the measurements shown in Supplementary Figure 6.

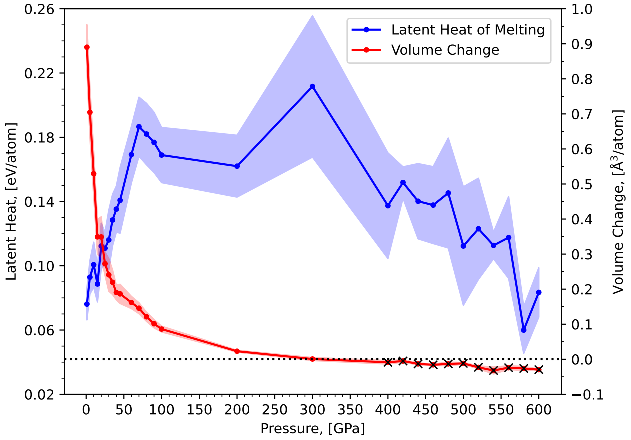

Supplementary Figure 6. **Volume changes and latent heats of melting for magnesium from 1-600 GPa.** Results are collected from the enthalpy curves produced by sampling the C4 O4 D14 ACE model and are shown in Supplementary Figure 7. Pale regions indicate the STD associated with the measurement, as explained in Supplementary Section VII, and the black crosses indicate negative thermal expansion.

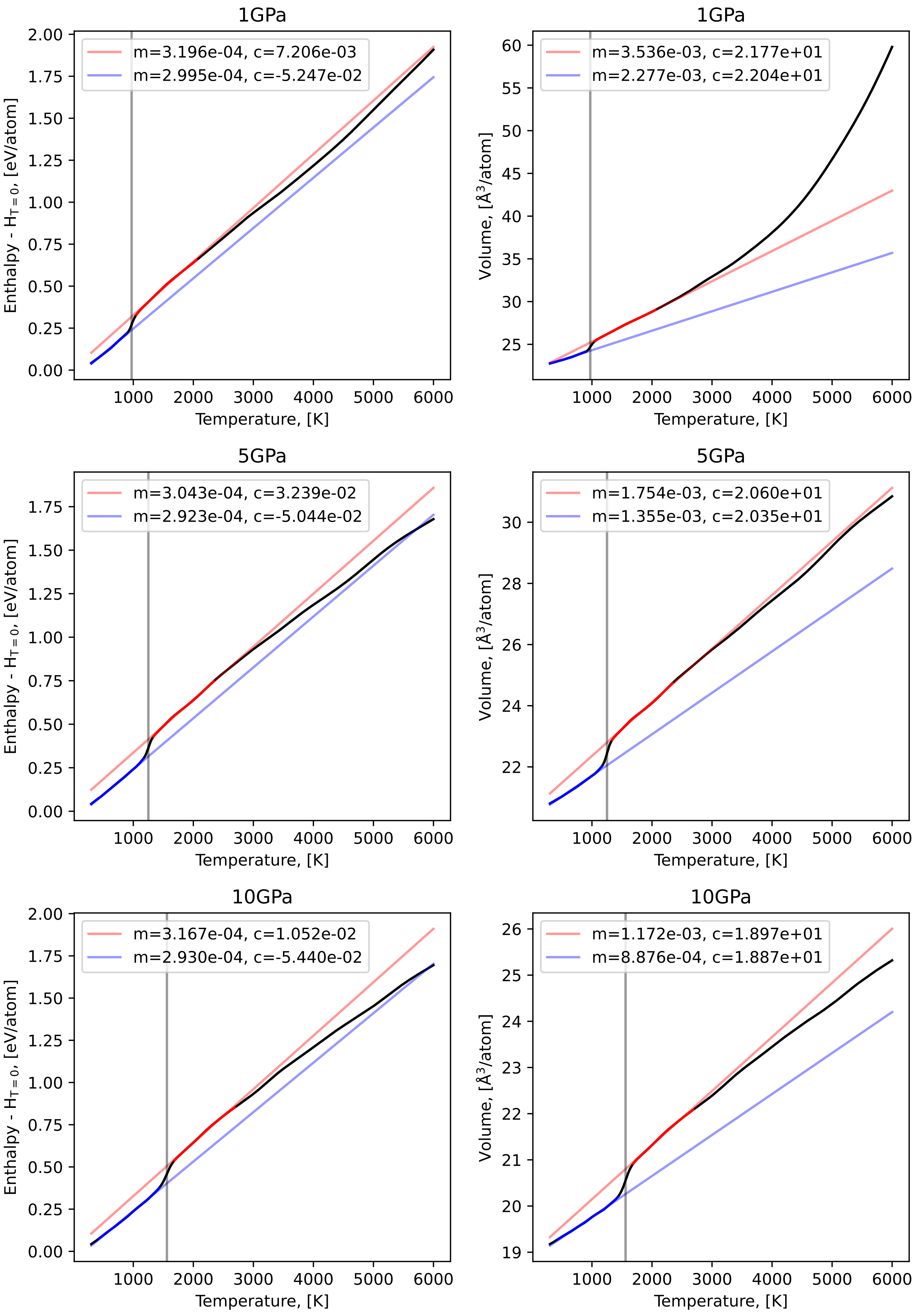
1GPa
m=3.196e-04, c=7.206e-03
m=2.995e-04, c=-5.247e-02
Enthalpy - $H_{T=0}$, [eV/atom]
Temperature, [K]
1GPa
m=3.536e-03, c=2.177e+01
m=2.277e-03, c=2.204e+01
Volume, [Å^3/atom]
Temperature, [K]
5GPa
m=3.043e-04, c=3.239e-02
m=2.923e-04, c=-5.044e-02
Enthalpy - $H_{T=0}$, [eV/atom]
Temperature, [K]
5GPa
m=1.754e-03, c=2.060e+01
m=1.355e-03, c=2.035e+01
Volume, [Å^3/atom]
Temperature, [K]
10GPa
m=3.167e-04, c=1.052e-02
m=2.930e-04, c=-5.440e-02
Enthalpy - $H_{T=0}$, [eV/atom]
Temperature, [K]
10GPa
m=1.172e-03, c=1.897e+01
m=8.876e-04, c=1.887e+01
Volume, [Å^3/atom]
Temperature, [K]

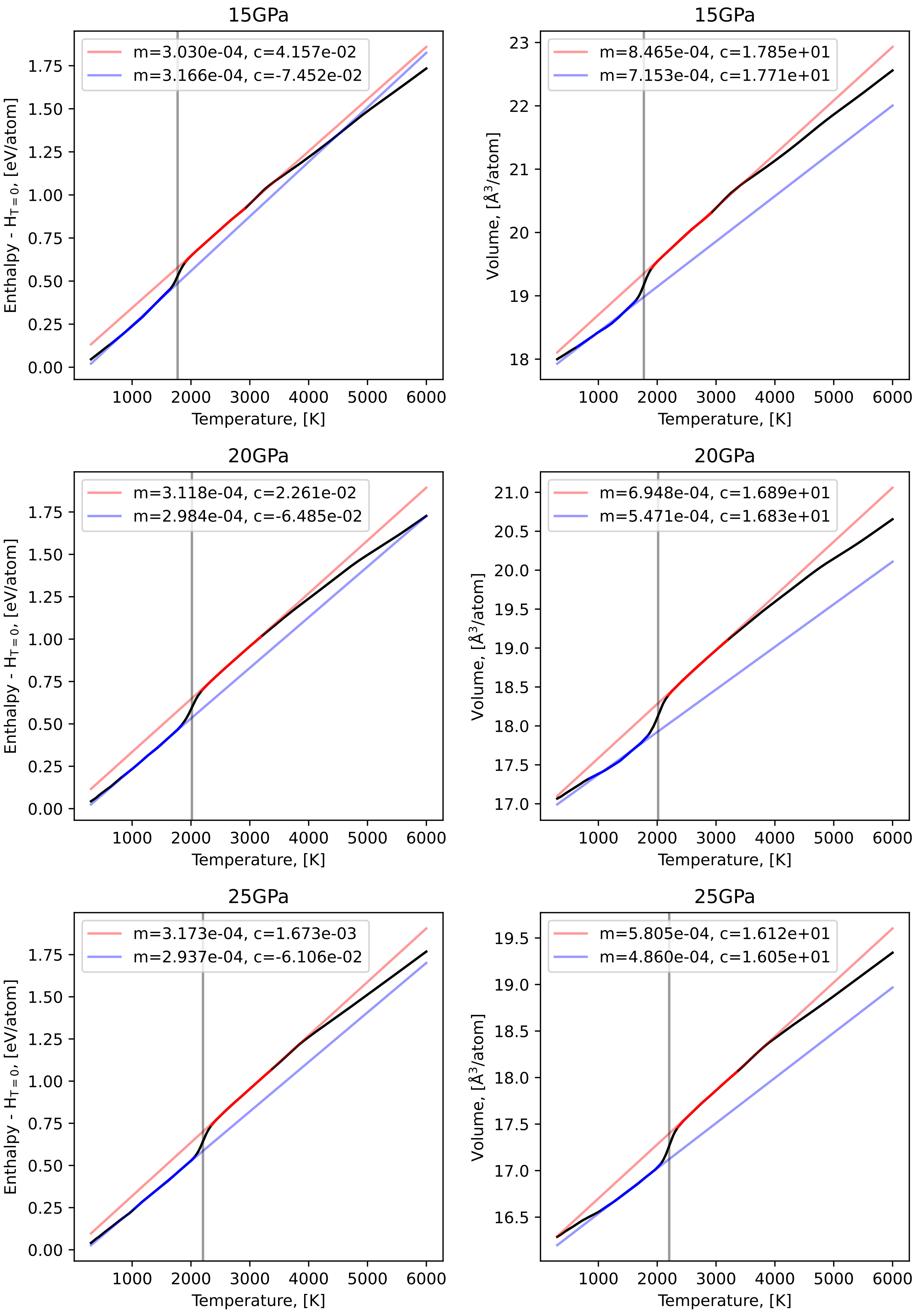
15GPa
m=3.030e-04, c=4.157e-02
m=3.166e-04, c=-7.452e-02
Enthalpy - $H_{T=0}$, [eV/atom]
Temperature, [K]
15GPa
m=8.465e-04, c=1.785e+01
m=7.153e-04, c=1.771e+01
Volume, [Å^3/atom]
Temperature, [K]
20GPa
m=3.118e-04, c=2.261e-02
m=2.984e-04, c=-6.485e-02
Enthalpy - $H_{T=0}$, [eV/atom]
Temperature, [K]
20GPa
m=6.948e-04, c=1.689e+01
m=5.471e-04, c=1.683e+01
Volume, [Å^3/atom]
Temperature, [K]
25GPa
m=3.173e-04, c=1.673e-03
m=2.937e-04, c=-6.106e-02
Enthalpy - $H_{T=0}$, [eV/atom]
Temperature, [K]
25GPa
m=5.805e-04, c=1.612e+01
m=4.860e-04, c=1.605e+01
Volume, [Å^3/atom]
Temperature, [K]

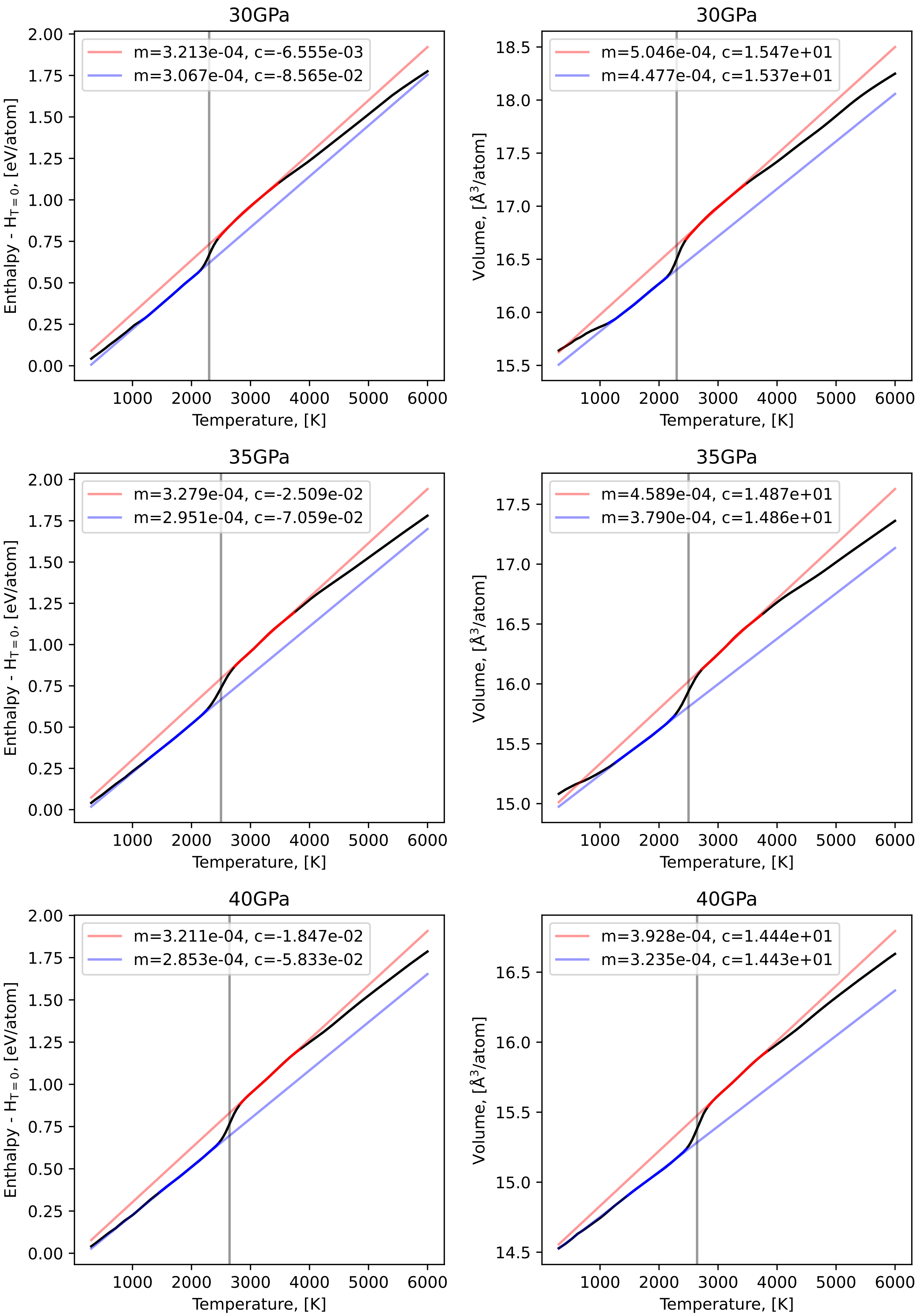
30GPa
m=3.213e-04, c=-6.555e-03
m=3.067e-04, c=-8.565e-02
Enthalpy - $H_{T=0}$, [eV/atom]
Temperature, [K]
30GPa
m=5.046e-04, c=1.547e+01
m=4.477e-04, c=1.537e+01
Volume, [Å^3/atom]
Temperature, [K]
35GPa
m=3.279e-04, c=-2.509e-02
m=2.951e-04, c=-7.059e-02
Enthalpy - $H_{T=0}$, [eV/atom]
Temperature, [K]
35GPa
m=4.589e-04, c=1.487e+01
m=3.790e-04, c=1.486e+01
Volume, [Å^3/atom]
Temperature, [K]
40GPa
m=3.211e-04, c=-1.847e-02
m=2.853e-04, c=-5.833e-02
Enthalpy - $H_{T=0}$, [eV/atom]
Temperature, [K]
40GPa
m=3.928e-04, c=1.444e+01
m=3.235e-04, c=1.443e+01
Volume, [Å^3/atom]
Temperature, [K]

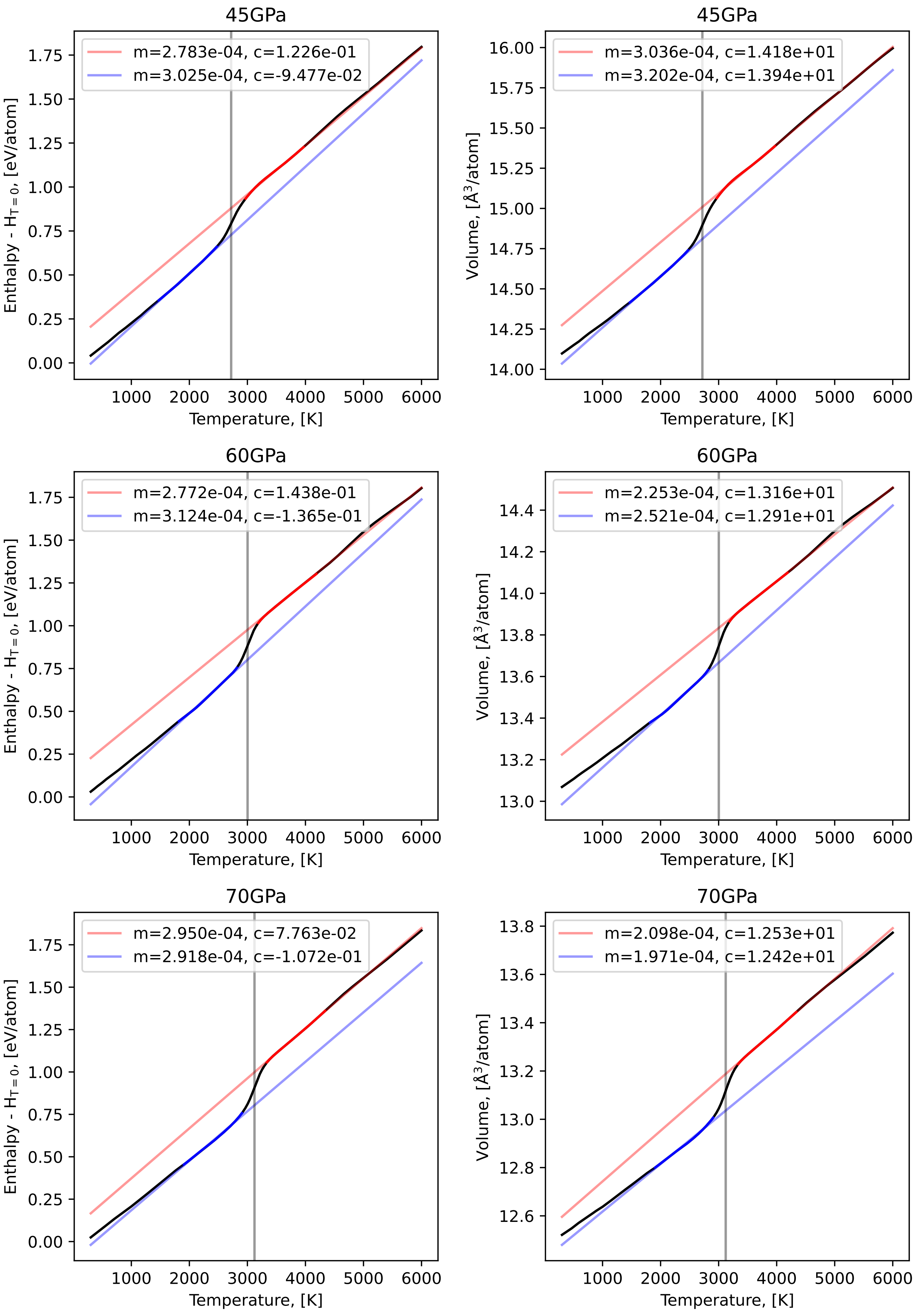
45GPa
m=2.783e-04, c=1.226e-01
m=3.025e-04, c=-9.477e-02
Enthalpy - $H_{T=0}$, [eV/atom]
Temperature, [K]
45GPa
m=3.036e-04, c=1.418e+01
m=3.202e-04, c=1.394e+01
Volume, [Å^3/atom]
Temperature, [K]
60GPa
m=2.772e-04, c=1.438e-01
m=3.124e-04, c=-1.365e-01
Enthalpy - $H_{T=0}$, [eV/atom]
Temperature, [K]
60GPa
m=2.253e-04, c=1.316e+01
m=2.521e-04, c=1.291e+01
Volume, [Å^3/atom]
Temperature, [K]
70GPa
m=2.950e-04, c=7.763e-02
m=2.918e-04, c=-1.072e-01
Enthalpy - $H_{T=0}$, [eV/atom]
Temperature, [K]
70GPa
m=2.098e-04, c=1.253e+01
m=1.971e-04, c=1.242e+01
Volume, [Å^3/atom]
Temperature, [K]

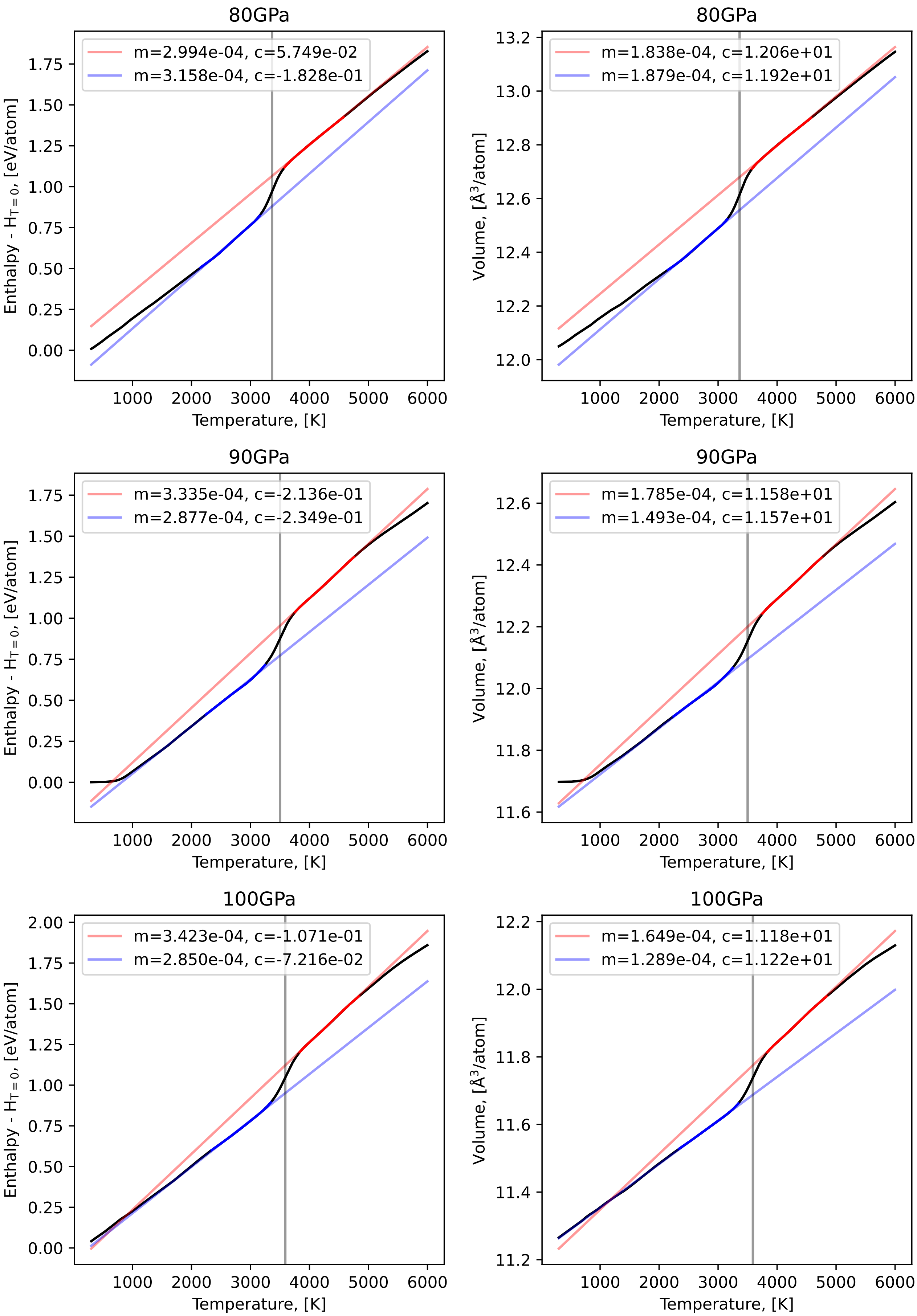
80GPa
m=2.994e-04, c=5.749e-02
m=3.158e-04, c=-1.828e-01
Enthalpy - $H_{T=0}$, [eV/atom]
Temperature, [K]
80GPa
m=1.838e-04, c=1.206e+01
m=1.879e-04, c=1.192e+01
Volume, [Å^3/atom]
Temperature, [K]
90GPa
m=3.335e-04, c=-2.136e-01
m=2.877e-04, c=-2.349e-01
Enthalpy - $H_{T=0}$, [eV/atom]
Temperature, [K]
90GPa
m=1.785e-04, c=1.158e+01
m=1.493e-04, c=1.157e+01
Volume, [Å^3/atom]
Temperature, [K]
100GPa
m=3.423e-04, c=-1.071e-01
m=2.850e-04, c=-7.216e-02
Enthalpy - $H_{T=0}$, [eV/atom]
Temperature, [K]
100GPa
m=1.649e-04, c=1.118e+01
m=1.289e-04, c=1.122e+01
Volume, [Å^3/atom]
Temperature, [K]

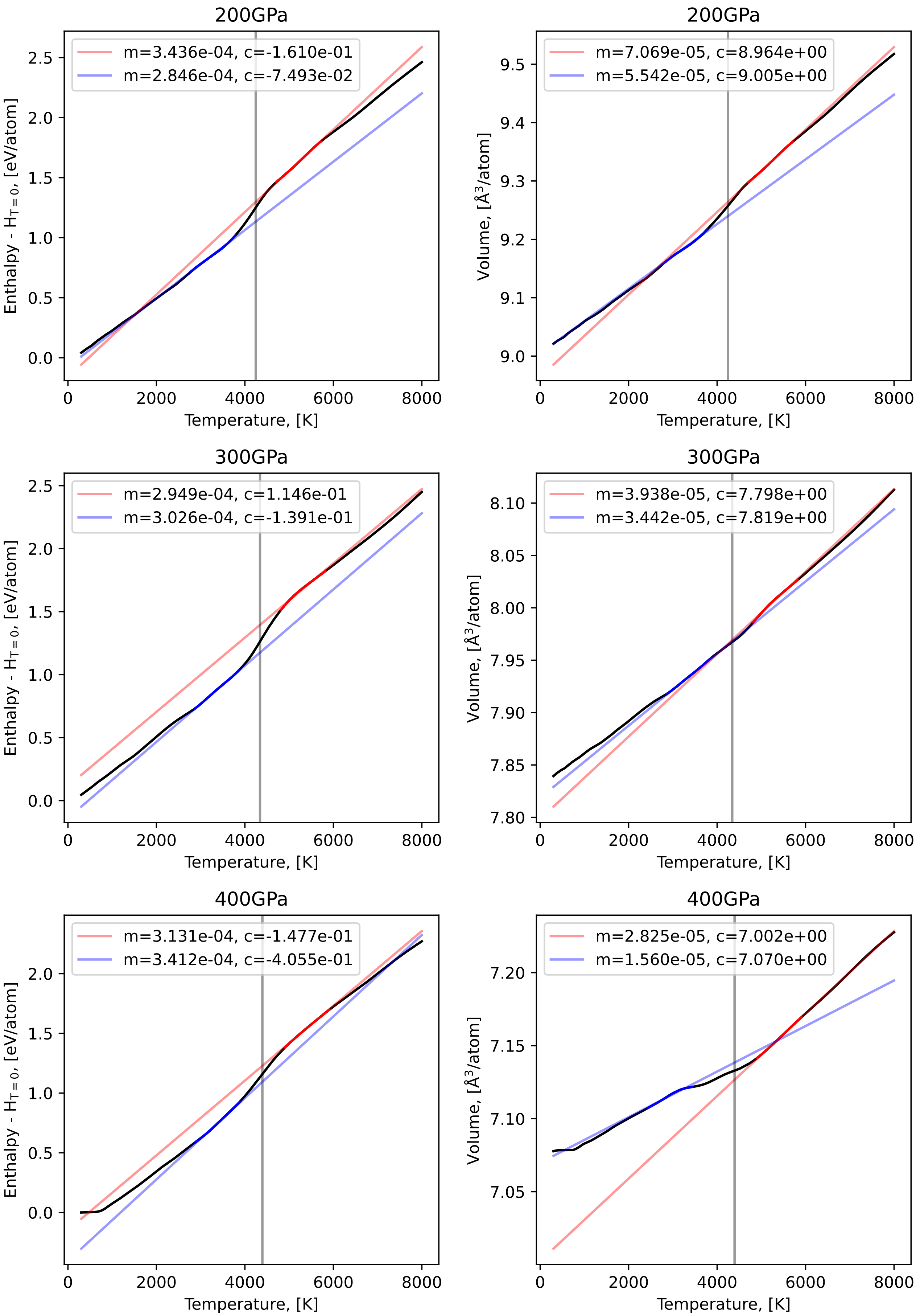
200GPa
m=3.436e-04, c=-1.610e-01
m=2.846e-04, c=-7.493e-02
Enthalpy - $H_{T=0}$, [eV/atom]
Temperature, [K]
200GPa
m=7.069e-05, c=8.964e+00
m=5.542e-05, c=9.005e+00
Volume, [Å^3/atom]
Temperature, [K]
300GPa
m=2.949e-04, c=1.146e-01
m=3.026e-04, c=-1.391e-01
Enthalpy - $H_{T=0}$, [eV/atom]
Temperature, [K]
300GPa
m=3.938e-05, c=7.798e+00
m=3.442e-05, c=7.819e+00
Volume, [Å^3/atom]
Temperature, [K]
400GPa
m=3.131e-04, c=-1.477e-01
m=3.412e-04, c=-4.055e-01
Enthalpy - $H_{T=0}$, [eV/atom]
Temperature, [K]
400GPa
m=2.825e-05, c=7.002e+00
m=1.560e-05, c=7.070e+00
Volume, [Å^3/atom]
Temperature, [K]

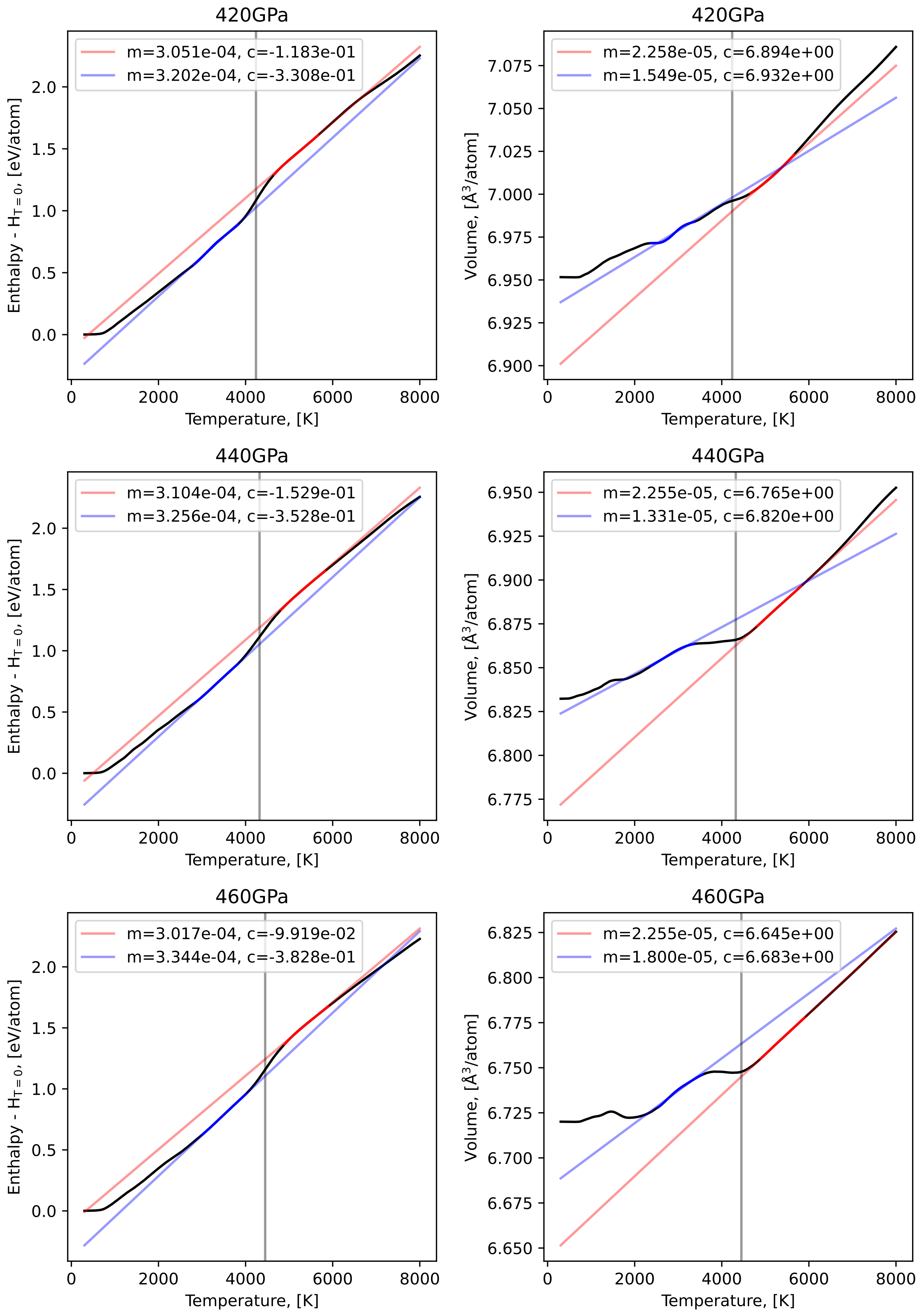
420GPa
m=3.051e-04, c=-1.183e-01
m=3.202e-04, c=-3.308e-01
Enthalpy - $H_{T=0}$, [eV/atom]
Temperature, [K]
420GPa
m=2.258e-05, c=6.894e+00
m=1.549e-05, c=6.932e+00
Volume, [Å^3/atom]
Temperature, [K]
440GPa
m=3.104e-04, c=-1.529e-01
m=3.256e-04, c=-3.528e-01
Enthalpy - $H_{T=0}$, [eV/atom]
Temperature, [K]
440GPa
m=2.255e-05, c=6.765e+00
m=1.331e-05, c=6.820e+00
Volume, [Å^3/atom]
Temperature, [K]
460GPa
m=3.017e-04, c=-9.919e-02
m=3.344e-04, c=-3.828e-01
Enthalpy - $H_{T=0}$, [eV/atom]
Temperature, [K]
460GPa
m=2.255e-05, c=6.645e+00
m=1.800e-05, c=6.683e+00
Volume, [Å^3/atom]
Temperature, [K]

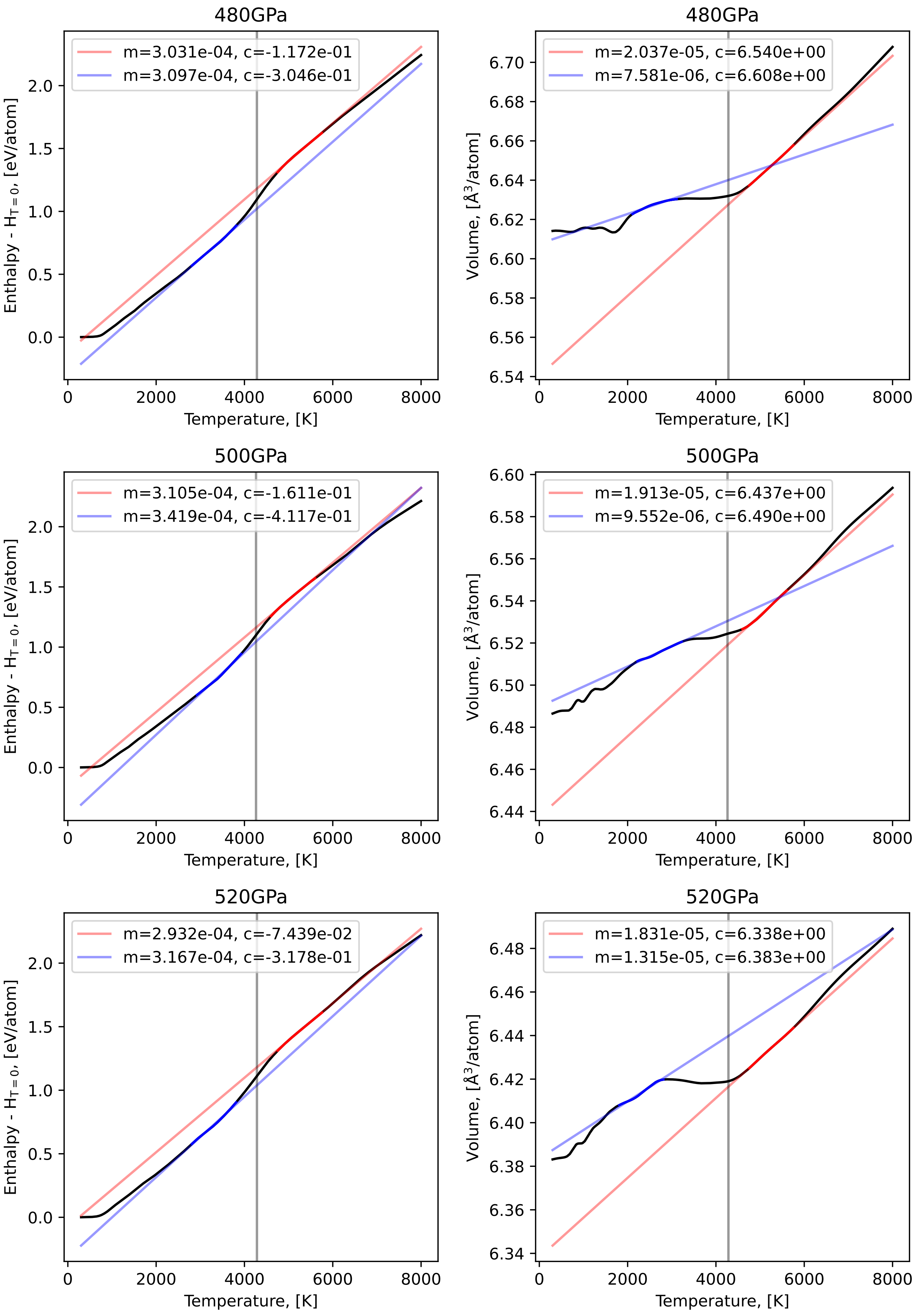
480GPa
m=3.031e-04, c=-1.172e-01
m=3.097e-04, c=-3.046e-01
Enthalpy - $H_{T=0}$, [eV/atom]
Temperature, [K]
480GPa
m=2.037e-05, c=6.540e+00
m=7.581e-06, c=6.608e+00
Volume, [Å^3/atom]
Temperature, [K]
500GPa
m=3.105e-04, c=-1.611e-01
m=3.419e-04, c=-4.117e-01
Enthalpy - $H_{T=0}$, [eV/atom]
Temperature, [K]
500GPa
m=1.913e-05, c=6.437e+00
m=9.552e-06, c=6.490e+00
Volume, [Å^3/atom]
Temperature, [K]
520GPa
m=2.932e-04, c=-7.439e-02
m=3.167e-04, c=-3.178e-01
Enthalpy - $H_{T=0}$, [eV/atom]
Temperature, [K]
520GPa
m=1.831e-05, c=6.338e+00
m=1.315e-05, c=6.383e+00
Volume, [Å^3/atom]
Temperature, [K]

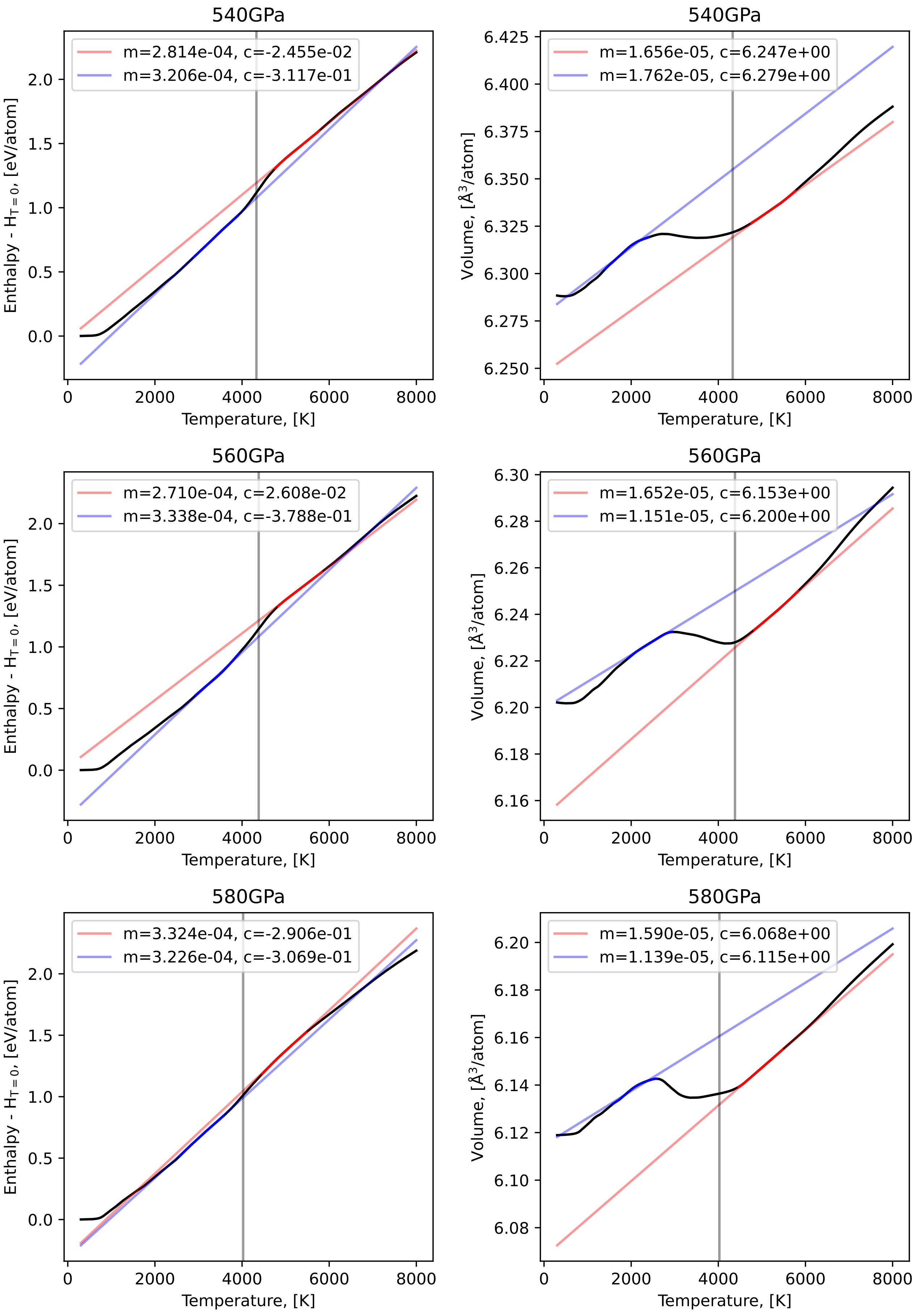
540GPa
m=2.814e-04, c=-2.455e-02
m=3.206e-04, c=-3.117e-01
Enthalpy - $H_{T=0}$, [eV/atom]
Temperature, [K]
540GPa
m=1.656e-05, c=6.247e+00
m=1.762e-05, c=6.279e+00
Volume, [Å^3/atom]
Temperature, [K]
560GPa
m=2.710e-04, c=2.608e-02
m=3.338e-04, c=-3.788e-01
Enthalpy - $H_{T=0}$, [eV/atom]
Temperature, [K]
560GPa
m=1.652e-05, c=6.153e+00
m=1.151e-05, c=6.200e+00
Volume, [Å^3/atom]
Temperature, [K]
580GPa
m=3.324e-04, c=-2.906e-01
m=3.226e-04, c=-3.069e-01
Enthalpy - $H_{T=0}$, [eV/atom]
Temperature, [K]
580GPa
m=1.590e-05, c=6.068e+00
m=1.139e-05, c=6.115e+00
Volume, [Å^3/atom]
Temperature, [K]

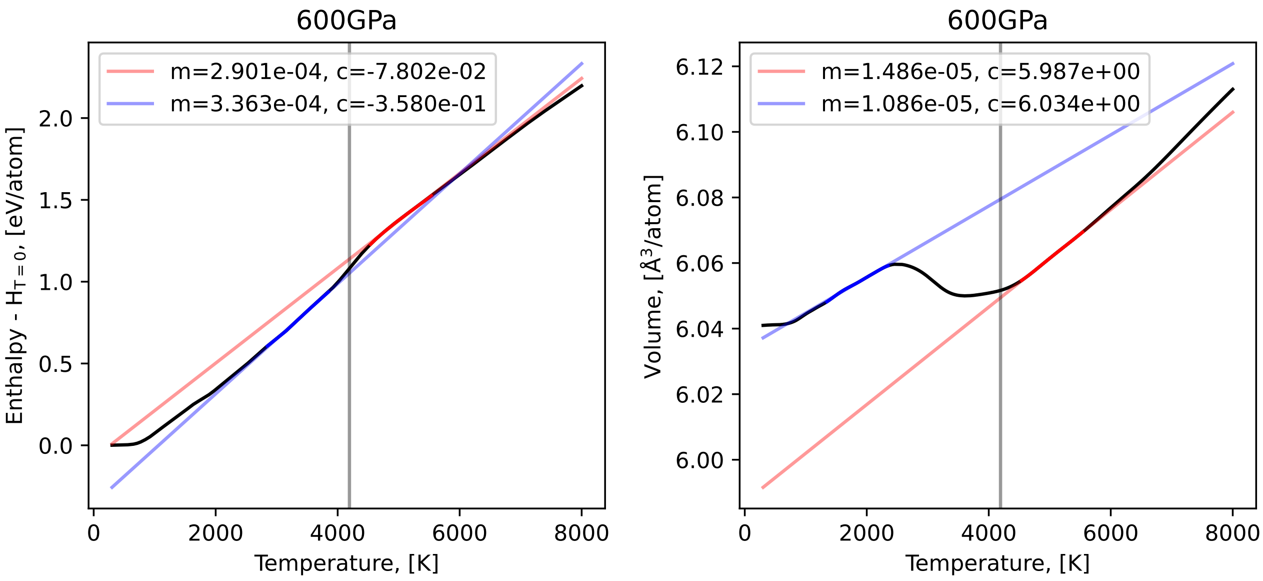

Supplementary Figure 7. **Temperature-enthalpy and temperature-volume plots produced from 64-atom NS using the C4 O4 D14 model at 1-600 GPa.** The transition temperatures are shown as vertical black lines. Linear functions were fitted to their respective coloured regions of the plots with the gradient, m, and y-intercept, c, given in the legend. This data was used to produce the results shown in Supplementary Figure 6 and calculate the thermal expansion coefficients shown in Main Figure 12.

[1] S. R. Wilson and M. I. Mendelev, A unified relation for the solid-liquid interface free energy of pure FCC, BCC, and HCP metals, The Journal of Chemical Physics **144**, 144707 (2016).

[2] G. W. Stinton, S. G. MacLeod, H. Cynn, D. Errandonea, W. J. Evans, J. E. Proctor, Y. Meng, and M. I. McMahon, Equation of state and high-pressure/high-temperature phase diagram of magnesium, Physical Review B **90**, 134105 (2014).

[3] E. Ibrahim, Y. Lysogorskiy, M. Mrovec, and R. Drautz, Atomic cluster expansion for a general-purpose interatomic potential of magnesium, Physical Review Materials **7**, 113801 (2023).

[4] L.-T. Li, X.-Y. Gao, H.-F. Liu, J.-W. Xian, F.-Y. Tian, and H.-F. Song, Multiphase equation of state for magnesium based on first-principles simulations, Physical Review B **110**, 224107 (2024).

[5] N. A. Smirnov, Comparative Analysis for the Behavior of Beryllium and Magnesium Crystals at Ultrahigh Pressures, Physica Status Solidi (b) **261**, 2300551 (2024).

[6] D. Errandonea, R. Boehler, and M. Ross, Melting of the alkaline-earth metals to 80 GPa, Physical Review B **65**, 012108 (2001).

[7] J. A. Moriarty and J. D. Althoff, First-principles temperature-pressure phase diagram of magnesium, Physical Review B **51**, 5609 (1995).

[8] S. Mehta, G. D. Price, and D. Alfè, Ab initio thermodynamics and phase diagram of solid magnesium: A comparison of the LDA and GGA, The Journal of Chemical Physics **125**, 194507 (2006).

[9] P. Li, G. Gao, Y. Wang, and Y. Ma, Crystal Structures and Exotic Behavior of Magnesium under Pressure, The Journal of Physical Chemistry C **114**, 21745 (2010).

[10] G. A. Marchant, M. A. Caro, B. Karasulu, and L. B. Pártay, Exploring the configuration space of elemental carbon with empirical and machine learned interatomic potentials, npj Computational Materials **9**, 1 (2023).

[11] K. Lejaeghere, G. Bihlmayer, T. Björkman, P. Blaha, S. Blügel, V. Blum, D. Caliste, I. E. Castelli, S. J. Clark, A. Dal Corso, S. de Gironcoli, T. Deutsch, J. K. Dewhurst, I. Di Marco, C. Draxl, M. Dułak, O. Eriksson, J. A. Flores-Livas, K. F. Garrity, L. Genovese, P. Giannozzi, M. Giantomassi, S. Goedecker, X. Gonze, O. Grånäs, E. K. U. Gross, A. Gulans, F. Gygi, D. R. Hamann, P. J. Hasnip, N. A. W. Holzwarth, D. Iuşan, D. B. Jochym, F. Jollet, D. Jones, G. Kresse, K. Koepernik, E. Küçükbenli, Y. O. Kvashnin, I. L. M. Locht, S. Lubeck, M. Marsman, N. Marzari, U. Nitzsche, L. Nordström, T. Ozaki, L. Paulatto, C. J. Pickard, W. Poelmans, M. I. J. Probert, K. Refson, M. Richter, G.-M. Rignanese, S. Saha, M. Scheffler, M. Schlipf, K. Schwarz, S. Sharma, F. Tavazza, P. Thunström, A. Tkatchenko, M. Torrent, D. Vanderbilt, M. J. van Setten, V. Van Speybroeck, J. M. Wills, J. R. Yates, G.-X. Zhang, and S. Cottenier, Reproducibility in density functional theory calculations of solids, Science **351**, aad3000 (2016).
